# Targeting Ferroptosis in Parkinson’s Disease: Mechanisms and Emerging Therapeutic Strategies

**DOI:** 10.3390/ijms252313042

**Published:** 2024-12-04

**Authors:** Minghao Zhou, Keyang Xu, Jianxian Ge, Xingnian Luo, Mengyao Wu, Ning Wang, Jianfeng Zeng

**Affiliations:** Center for Molecular Imaging and Nuclear Medicine, State Key Laboratory of Radiation Medicine and Protection, School for Radiological and Interdisciplinary Sciences (RAD-X), Collaborative Innovation Center of Radiological Medicine of Jiangsu Higher Education Institutions, Soochow University, Suzhou 215123, China; 2102402005@stu.suda.edu.cn (M.Z.); 20224220040@stu.suda.edu.cn (K.X.); 2230507024@stu.suda.edu.cn (X.L.); 20234220047@stu.suda.edu.cn (M.W.); 20234020010@stu.suda.edu.cn (N.W.)

**Keywords:** Parkinson’s disease, ferroptosis, ferroptosis inhibitors, oxidative stress

## Abstract

Parkinson’s disease (PD) is a common neurodegenerative disorder characterized by the loss of dopaminergic neurons in the substantia nigra and the accumulation of α-synuclein in the brain. Ferroptosis, a recently identified form of regulated cell death, is critical in PD pathogenesis due to its association with iron deposition, overproduction of reactive oxygen species, iron-dependent lipid peroxidation and impaired lipid peroxidation clearance. This cell death mechanism is closely linked to several pathogenic processes in PD, including α-synuclein aggregation, oxidative stress, mitochondrial dysfunction, microglia-induced neuroinflammation, and neuromelanin accumulation. Given the significant role of ferroptosis in these mechanisms, there is increasing interest in targeting ferroptosis for PD treatment. Several drugs have shown potential in alleviating PD symptoms by inhibiting ferroptosis. This review aims to consolidate current knowledge on ferroptosis in PD and assess the therapeutic potential of anti-ferroptosis drugs, highlighting promising directions for future research and clinical applications.

## 1. Introduction

Parkinson’s disease (PD) is a prevalent neurodegenerative disorder, primarily affecting the elderly. As of 2017, the incidence rate of PD among individuals over 60 years old is approximately 1% [1]. PD manifests with both motor dysfunctions, such as resting tremor, and non-motor symptoms, including depression, fatigue, autonomic dysfunction, and sleep disturbances [2]. These symptoms significantly impair the quality of life of PD patients, attracting considerable societal attention.

The primary pathological features of PD include the degeneration and death of dopaminergic neurons in the substantia nigra (SN) and the accumulation of the pathological protein α-synuclein (α-syn). Current clinical treatments mainly comprise levodopa therapy, surgical interventions like pallidotomy, and deep brain stimulation. However, long-term use of levodopa leads to complications such as dyskinesia, motor fluctuations, and impulse control disorders (ICD), largely due to its poor blood–brain barrier (BBB) penetration, necessitating high doses [3,4]. Moreover, levodopa fails to halt disease progression. Surgical treatments, while effective, pose significant physical risks. Therefore, developing new therapeutic strategies that can fundamentally prevent PD progression is crucial.

Ferroptosis, a newly identified form of cell death distinct from apoptosis, necrosis, and autophagy has emerged as a potential target in PD therapy due to its involvement in PD pathogenesis [5]. This form of cell death is characterized by iron accumulation, overproduction of reactive oxygen species (ROS), iron-dependent lipid peroxidation (LPO), and impaired LPO clearance. Fe(II) plays a pivotal role in ferroptosis by generating substantial amounts of ROS through the Fenton reaction, while Fe(III) catalyzes the conversion of superoxide radicals and hydrogen peroxide into hydroxyl radicals and oxygen via the Haber–Weiss reaction, thereby exacerbating oxidative damage [5]. Concurrently, ROS attack polyunsaturated fatty acids (PUFAs), converting them into LPO [6]. The accumulation of LPO ultimately induces cell death [7]. Additionally, during ferroptosis, the antioxidant capacities of the System Xc^−^-GPX4/GSH pathway, the FSP1/CoQ_10_ pathway, the GCH1-BH4/BH2 pathway, and other antioxidant pathway are compromised [8,9,10]. This decrease in antioxidant function further aggravates oxidative damage and contributes to cell death.

Recent research indicates that ferroptosis may play a crucial role in the pathogenesis of PD [6]. Studies have shown that the iron content in the SN of PD patients is significantly higher than in healthy individuals [11]. Additionally, levels of PUFAs are decreased, while levels of the lipid peroxide intermediate malondialdehyde (MDA) are increased in the SN of PD patients [12]. These findings suggest impaired LPO clearance and elevated ROS levels are hallmarks of ferroptosis, thereby establishing a strong correlation between ferroptosis and PD. Various in vitro and in vivo experiments have identified a series of drugs that inhibit the death of dopaminergic neurons in the SN through anti-ferroptosis mechanisms. This review aims to summarize the mechanisms by which ferroptosis contributes to the onset and progression of PD and to evaluate the therapeutic potential of anti-ferroptosis drugs. By doing so, it seeks to provide valuable insights for future research and the development of treatments for PD.

## 2. The Role of Ferroptosis in PD Pathogenesis

**Figure 1:**
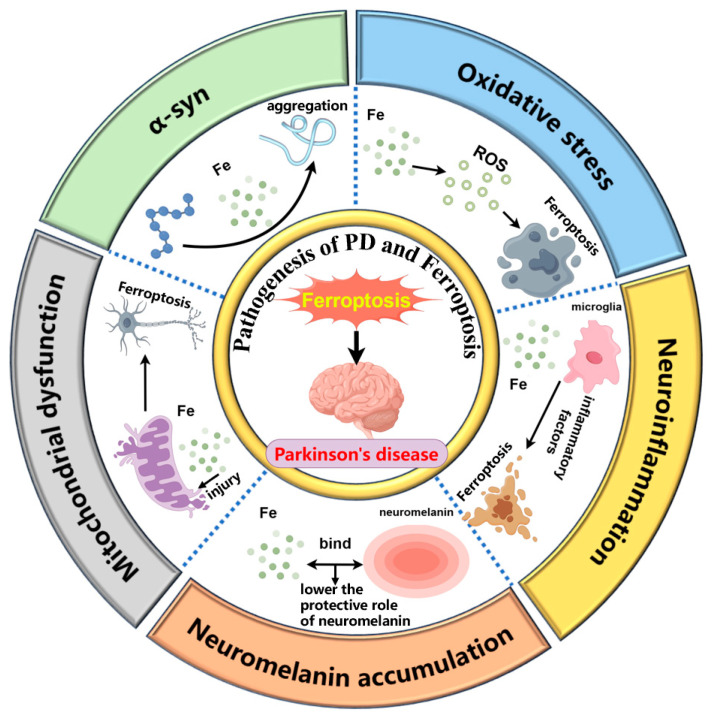
The interplay between ferroptosis and key pathological mechanisms in Parkinson’s disease, including α-synuclein aggregation, oxidative stress, mitochondrial dysfunction, microglia-induced neuroinflammation, and neuromelanin accumulation. (1) Iron promotes the pathological aggregation of α-synuclein. (2) Iron contributes to ferroptosis by generating ROS. (3) Iron damages mitochondria, triggering ferroptosis. (4) Iron activates microglia, leading to the release of inflammatory factors that exacerbate ferroptosis. (5) Excess iron binds to neuromelanin, reducing its neuroprotective role. By Figdraw 2.0.

The pathogenesis of PD is believed to involve several key mechanisms, including the accumulation of α-syn in the brain [13], oxidative stress [14], mitochondrial dysfunction [15], neuroinflammation caused by microglia activation [16], and neuromelanin (NM) accumulation [17]. Ferroptosis is closely associated with these mechanisms. The relationship between various pathogenesis of PD and ferroptosis is shown in Figure 1.

### 2.1. α-syn and Ferroptosis

α-syn is considered a key protein in the pathogenesis of PD due to its significant roles in oxidative stress, mitochondrial function regulation, and neuroinflammation [18]. Studies have shown a close relationship between ferroptosis and α-syn aggregation.

Iron promotes α-syn aggregation in the brain. Both in vivo and in vitro studies have demonstrated that α-syn rapidly accumulates upon exposure to iron [19]. Fe(III) can directly and weakly bind to the C-terminal of α-syn, promoting its pathological aggregation at low concentrations [20]. Additionally, there are iron response elements (IREs) on the 5′-untranslated region (UTR) of α-syn mRNA, which can bind to iron regulatory proteins (IRPs). When bound, these IRPs inhibit the translation of α-syn mRNA. Iron can reverse this inhibition, thereby regulating α-syn at the translational level [21]. Moreover, α-syn is extensively phosphorylated in Lewy bodies [22]. Phosphorylated α-syn has been shown to exert toxic effects on cells [23], and iron accumulation within cells may promote α-syn phosphorylation by modulating kinase expression, thereby establishing a connection between iron and α-syn modification [24].

Conversely, α-syn can influence ferroptosis through various pathways. The iron reductase activity of α-syn can reduce Fe(III) to Fe(II) [25]. Increased α-syn expression in neurons may elevate Fe(II) levels, leading to ROS production via the Fenton reaction, resulting in neurotoxicity. The *SNCA* gene, which encodes α-syn, has been shown to reduce cell activity when overexpressed. The use of ferroptosis inhibitors such as deferoxamine (DFO, an iron chelator), deuterated PUFAs, and Ferrostatin-1 (Fer-1) significantly reduces α-syn oligomer-induced cell death, restoring normal cell function. Therefore, α-syn oligomers can induce ferroptosis, while ferroptosis inhibitors can prevent α-syn-induced cell death [26]. Iron and α-syn may cause neurotoxicity synergistically [26].

Furthermore, the relationship between α-syn and LPO production is noteworthy. In SH-SY5Y cells overexpressing α-syn, elevated levels of propanoylated lysine, a specific indicator of PUFAs oxidation, have been observed. This increase may result from α-syn-induced ROS production, which promotes neuronal LPO and subsequent cell death [27]. Additionally, α-syn has been shown to enhance cellular sensitivity to LPO and ferroptosis in neural precursor cells overexpressing α-syn [28]. Products of PUFAs peroxidation, such as 4-HNE, can modify α-syn, causing its oligomerization and pathological accumulation [29]. Furthermore, ROS and LPO generated during ferroptosis can impair proteasome function, further exacerbating α-syn aggregation [30]. These findings suggest a synergistic relationship between α-syn and LPO, thereby promoting ferroptosis and causing neurotoxicity.

### 2.2. Oxidative Stress and Ferroptosis

The defense mechanisms against ferroptosis primarily involve three critical pathways: the System Xc^−^-GPX4/GSH pathway, the FSP1/CoQ_10_ pathway, and the GCH1-BH4/BH2 pathway. Among these, the System Xc^−^-GPX4/GSH pathway is the most essential. In both PD patients and PD animal models, all three pathways are inhibited, contributing to oxidative stress and neuronal vulnerability.

The System Xc^−^-GPX4/GSH pathway is vital in defending against ferroptosis. GPX4 is a key enzyme that catalyzes the reduction of LPO into non-toxic lipid alcohols using glutathione (GSH) as a cofactor. In the SN of PD patients, numerous studies have demonstrated that free iron content and LPO levels are elevated, while GSH levels are significantly reduced, which contributes to heightened oxidative damage and ferroptosis [31]. The System Xc^−^, a cystine/glutamate antiporter comprising SLC7A11 and SLC3A2, is essential for maintaining intracellular GSH levels. It facilitates the transport of extracellular cystine and intracellular glutamate in a 1:1 ratio. Once inside the cell, cystine is converted into cysteine, a precursor for GSH synthesis. Reduced expression of the SLC7A11 gene in PD patients impairs System Xc^−^ activity, leading to decreased cystine uptake and subsequent GSH depletion [32]. Once transported into the cell, cystine is converted into cysteine, which combines with glutamate to form γ-glutamylcysteine, catalyzed by γ-glutamylcysteine ligase (GCL). Subsequently, γ-glutamylcysteine and glycine are converted into GSH by GSH synthetase (GS). Because cysteine availability is the rate-limiting step in GSH synthesis, the activity of System Xc^−^ is indispensable in regulating intracellular GSH levels [33]. The protein SLC1A5 located on the membrane can transport glutamine into the cell and transform into glutamate under the action of Glutaminases (GLS), which can also promote the production of GSH [34].

In addition, intracellular cysteine levels are regulated by DJ-1 through the trans-sulfuration pathway, which serves as a bypass for cysteine synthesis. In this pathway, methionine is converted into S-adenosyl homocysteine (SAH) via the methionine cycle. SAH is then hydrolyzed into homocysteine (Hcy) by S-adenosyl homocysteine hydrolase (SAHH). Under the action of vitamin B_6_-dependent enzymes cystathionine-β-synthase (CBS) and cystathionine-γ-lyase, Hcy is further converted into cysteine. Importantly, adenosyl homocysteinase-like 1 (AHCYL-1) acts as a potential inhibitor of SAHH, and DJ-1 suppresses AHCYL-1 to maintain this pathway’s activity. When DJ-1 is inactivated, AHCYL-1 inhibits SAHH, thereby disrupting the trans-sulfuration pathway and reducing cysteine synthesis. Thus, DJ-1 plays a critical role in regulating intracellular cysteine levels. Evidence suggests that suppression of DJ-1 enhances ferroptosis in vivo [35]. Furthermore, mutations in the DJ-1 gene (*PARK7*) increase mitochondrial oxidative stress, contributing to autosomal recessive early-onset PD.

CoQ_10_ and its reduced form, CoQ_10_-H_2_, are potent mitochondrial and LPO antioxidants, so CoQ_10_-H_2_ is also considered an inhibitor of ferroptosis. This reduction process is catalyzed by ferroptosis suppressor protein 1 (FSP1), located on the plasma membrane [9]. In addition, dihydroorotate dehydrogenase (DHODH) on the inner membrane of the mitochondria catalyzes CoQ_10_ into CoQ_10_-H_2_ [36]. The mevalonate (MVA) pathway also produces CoQ_10_ through a series of enzymatic reactions [37]. However, CoQ_10_ levels are reduced in both PD animal models and patients [9], weakening the antioxidant capacity of the CoQ_10_ system and increasing susceptibility to ferroptosis. Another important antioxidant pathway in ferroptosis involves the GCH1-BH4/BH2-phospholipid axis. GTP cyclohydrolase-1 (GCH1) and its metabolic products, tetrahydrobiopterin (BH4) and dihydrobiopterin (BH2), form this axis, with BH4 serving as an endogenous antioxidant that protects against ferroptosis. This axis also regulates CoQ_10_ abundance, further enhancing ferroptosis resistance. BH4 likely increases the conversion of phenylalanine to tyrosine, which is subsequently transformed into 4-hydroxybenzoate, a precursor to CoQ_10_ [38]. However, postmortem analyses of PD patient tissues reveal significantly reduced BH4 levels and GCH1 activity in the SN and striatum, impairing resistance to oxidative stress [10]. Moreover, mutations in the GCH1 gene have been associated with a heightened risk of developing PD [39,40]. Therefore, elevated oxidative stress is closely linked to ferroptosis and contributes significantly to the pathogenesis of PD.

### 2.3. Mitochondrial Dysfunction and Ferroptosis

Mitochondria are essential organelles responsible for cellular energy production. Mitochondrial dysfunction is a key mechanism in the pathogenesis of PD and is closely related to ferroptosis. Mitochondrial morphological characteristics can be observed during ferroptosis, including reduced or absent mitochondrial ridges, increased mitochondrial membrane density, and ruptured mitochondrial outer membranes [41]. These features indicate mitochondrial damage during the development of ferroptosis.

Mitochondrial DNA is particularly vulnerable to damage, and iron deposition can lead to mitochondrial DNA breakage or loss. This results in reduced mitochondrial DNA transcription and decreased expression of respiratory chain subunits [42]. Additionally, iron oxidizes dopamine to form 6-hydroxydopamine (6-OHDA) [43], which can cause mitochondrial dysfunction by inhibiting Complexes I and IV. Mitochondrial ferritin, closely related to iron metabolism, indicates that mitochondrial dysfunction may cause iron metabolism disorders [44].

1-methyl-4-phenyl-1,2,3,6-tetrahydropyridine (MPTP) is a mitochondrial toxin that, upon crossing the BBB, is converted to 1-methyl-4-phenylpyridinium ion (MPP^+^). MPP^+^ inhibits Complex I, causing mitochondrial dysfunction and is commonly used to establish PD models in vivo and in vitro. In MPTP-induced mouse PD models, ferroptosis is implicated in dopaminergic neuron death, and this toxicity can be inhibited by the ferroptosis-specific inhibitor Fer-1 [45]. Similarly, rotenone, another mitochondrial toxin that inhibits Complex I, can induce PD in rat models. Idebenone, a CoQ_10_ analogue, has been shown to alleviate dyskinesia symptoms in these PD rat models [46].

**Figure 2:**
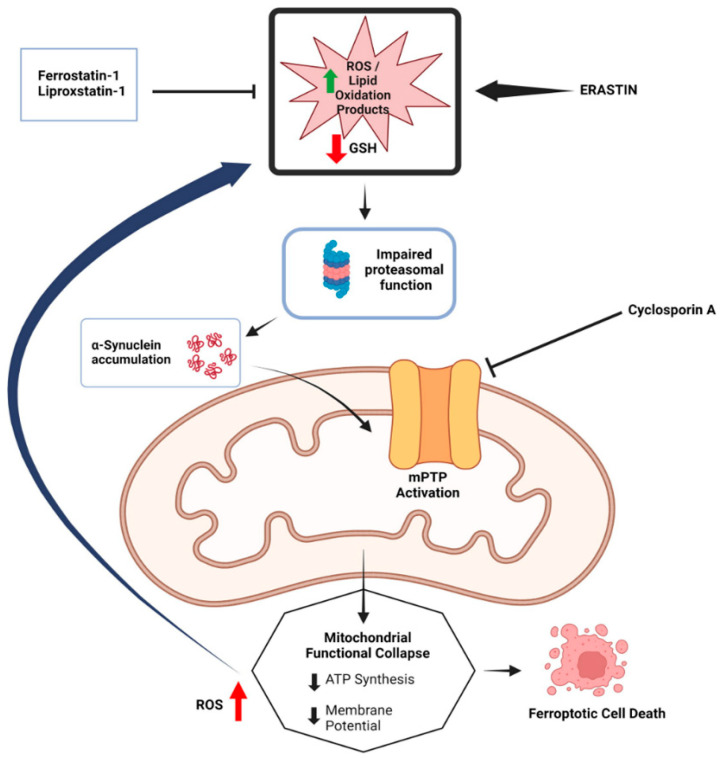
The potential relationship between α-synuclein accumulation and mitochondrial dysfunction in ferroptosis. The diagram illustrates how the accumulation of α-synuclein, along with impaired proteasomal function and mitochondrial permeability transition pore (mPTP) activation, leads to mitochondrial functional collapse, characterized by reduced ATP synthesis and membrane potential. This collapse, exacerbated by increased ROS and lipid peroxidation products, ultimately results in ferroptotic cell death. Inhibitors such as Fer-1 and Cyclosporin A, are shown to mitigate these effects by preventing ROS accumulation and protecting mitochondrial integrity. (Reprinted with permission from Ref. [30]. Copyright 2024, Taylor and Francis).

The interaction between α-syn and mitochondria is noteworthy in the context of ferroptosis. Erastin-induced ferroptosis in SH-SY5Y cells results in oxidative stress marker accumulation, the loss of mitochondrial membrane potential, and decreased intracellular ATP levels. This is primarily attributed to increased mitochondrial membrane permeability caused by the binding of erastin to voltage-dependent anion channel 2 (VDAC2) and VDAC3 on the mitochondrial outer membrane. This interaction disrupts NADH oxidation, reduces GSH production, and facilitates the generation and release of ROS [47]. Interestingly, α-syn can also bind to VDAC, impairing ATP/ADP transport and interfering with mitochondrial respiration through VDAC-mediated mechanisms [48]. Additionally, α-syn may interact with the mitochondrial permeability transition pore (mPTP), further contributing to ferroptosis [30]. These findings suggest that α-syn interacts with several mitochondrial membrane proteins, exacerbating mitochondrial dysfunction and promoting ferroptosis. The possible relationship between α-syn and mitochondria in ferroptosis is shown in the Figure 2.

### 2.4. Microglia Associated Neuroinflammation and Ferroptosis

Microglia are phagocytes in the central nervous system that release cytokines and engulf apoptotic neurons. The SN contains a large number of microglia compared to other central nervous system structures. Under normal circumstances, microglia play a role in preventing PD by performing functions such as phagocytosing apoptotic neurons. They also establish connections with neurons through tunneling nanotubes (TNTs) to extract α-syn from them and deliver healthy mitochondria [49]. However, in pathological conditions, microglia become one of the initiators of neuroinflammation [16]. Studies have shown extensive multiplication of reactive microglia in the SN of PD patients. These microglia shift from an early anti-inflammatory phenotype to a pro-inflammatory phenotype [50], inducing the loss of dopaminergic neurons through the production of inflammatory factors [51]. Activated microglia can release inflammatory factors and increase iron deposition in neurons and themselves by up-regulating Divalent Metal Transporter 1 (DMT1) and down-regulating ferroportin 1 (FPN1) expression [52]. Microglia are also sensitive to iron deposition, which significantly alters their transcriptional state, leading to the production and release of cytokines. This environment enhances the lipid peroxidation process in surrounding neurons, resulting in neurotoxicity and ultimately inducing PD [53]. The above evidence indicates a synergistic interaction between ferroptosis and microglia-induced neuroinflammation, contributing to the pathogenesis of PD.

### 2.5. NM Accumulation and Ferroptosis

Studies have shown that NM has both protective and damaging effects on neurons. On the one hand, NM can bind with iron to form an iron-NM complex, which inhibits iron-induced oxidative stress [54]. On the other hand, NM can act as an endogenous activator of microglia, leading to neuroinflammation and selective toxicity to dopaminergic neurons [17]. Additionally, NM can enhance the toxic effects of α-syn [55]. However, the oxidative degradation of NM increases with elevated iron load [54]. This evidence suggests a competitive relationship between ferroptosis and NM-induced dopaminergic neuron death. Under non-pathological conditions, NM primarily exerts protective effects on neurons. However, intracellular iron deposition may increase cell sensitivity to ferroptosis by weakening NM’s protective effects.

In summary, ferroptosis plays a crucial role in the pathogenesis and progression of PD. This form of cell death, distinct from apoptosis, necrosis, and autophagy, is characterized by iron accumulation, overproduction of ROS, iron-dependent LPO and impaired LPO clearance. These mechanisms are closely intertwined with several pathogenic processes in PD, making ferroptosis a significant contributor to neuronal degeneration. Inhibiting ferroptosis may, therefore, become a valuable target for the treatment of PD.

## 3. Therapeutic Approaches Targeting Ferroptosis in PD

Given the role of ferroptosis in the pathogenesis and progression of PD, numerous drugs are currently under research, with some having progressed to clinical trials and shown promising results. These anti-ferroptosis drugs may help alleviate or treat PD by protecting dopaminergic neurons from ferroptotic cell death.

### 3.1. Decreasing Intracellular Iron Load

**Figure 3:**
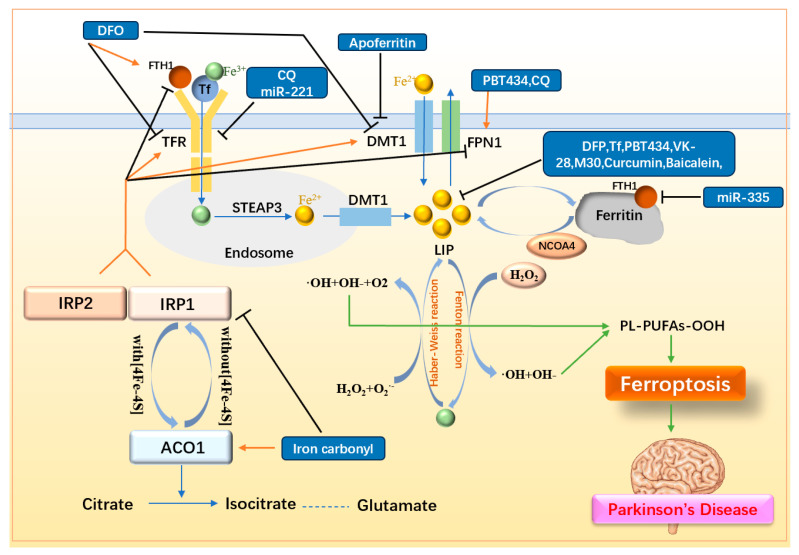
Mechanisms of iron metabolism and related drugs. Fe(II) in the LIP interconverts with Fe(III), generating ROS via the Fenton and Haber–Weiss reactions, thereby promoting LPO and triggering ferroptosis. Upregulation of iron storage proteins (FTH1, Tf), downregulation of iron transporters (TFR, DMT1), upregulation of the iron efflux transporter FPN1, and suppression of IRP1/2 collectively reduce intracellular iron levels and LIP, thereby inhibiting ferroptosis. FTH1: ferritin H chain; Tf: transferrin; TFR: transferrin receptor; DMT1: Divalent Metal Transporter; FPN1: ferroportin 1; LIP: labile iron pool; STEAP3: ferrireductase; NCOA4: nuclear receptor coactivator 4; ACO1: aconitase 1; IRP1/2: iron regulatory protein 1/2; DFO: deferoxamine; DFP: deferiprone; CQ: clioquinol.

As Fe(II) and Fe(III) contribute to ROS production through the Fenton and Haber–Weiss reactions, respectively, iron is considered one of the core driving factors of ferroptosis. Thus, reducing intracellular iron levels is crucial for preventing ferroptosis. Iron homeostasis within cells is maintained through a complex interplay involving iron storage proteins, transporters, and regulatory proteins. Ferritin, the primary iron storage protein, is composed of ferritin H chain (FTH) and ferritin L chain (FTL), with FTH playing the dominant role in iron storage [56]. FTH1 has also been implicated in ferritinophagy, where its reduction leads to increased levels of nuclear receptor coactivator 4 (NCOA4). This, in turn, promotes ferritin degradation, resulting in elevated intracellular iron levels [57]. Iron transporters include transferrin receptor (TFR), DMT1, and FPN1. TFR facilitates the uptake of iron into cells by binding transferrin-bound Fe(III) and ferritin. Within endosomes, Fe(III) is reduced to Fe(II) by STEAP3, a ferrireductase enzyme [58]. DMT1 is another critical iron transporter that contributes to non-heme iron uptake in most cell types [59]. DMT1 also exists on endosome and is responsible for transporting Fe(II) to the labile iron pool (LIP) within the cell. FPN1, on the other hand, reduces intracellular iron by transporting iron outside the cell. Iron regulatory proteins include IRP1 and IRP2. The levels of IRP2 are tightly regulated by iron- and oxygen-dependent proteasomal degradation, and are stabilized under low iron conditions. IRP1 exhibits dual functions, acting as either aconitase 1 (ACO1) or as an RNA-binding protein, depending on the presence of the [4Fe-4S] cluster. ACO1 converts citrate into isocitrate, a precursor for glutamate production. Both IRP1 and IRP2 bind to IREs on mRNA to regulate the expression of key iron-related proteins. Specifically, IRPs increase the expression of TFR and DMT1 while decreasing the expression of FPN1 and FTH1, thereby elevating the intracellular LIP [60]. The regulation of iron homeostasis and the mechanisms of iron-targeting drugs in ferroptosis in PD are summarized in Figure 3 and listed in Table 1.

#### 3.1.1. Simple Iron Chelators

DFO was one of the first iron chelators used in the treatment of PD. Studies have shown that DFO alleviates motor deficits in MPTP-induced PD mouse models and increases the survival rate of TH-positive neurons [61]. DFO also reduces the number of iron-positive cells in the SN and striatum, and downregulates the expression of α-syn, DMT1, and TFR. In PC12 cells induced by nerve growth factor (NGF), DFO was also found to alter the expression of FTH1, GPX4, and acyl CoA synthase long-chain family member 4 (ACSL4) [62]. However, its clinical application is limited due to its poor ability to cross the BBB. Deferiprone (DFP), an FDA-approved oral iron chelator, can cross the BBB and is thus favored for PD treatment [63]. In MPTP-induced PD mouse models, DFP has been shown to increase GSH levels, reduce mitochondrial LIP, and inhibit MPTP-induced dopamine depletion in the striatum. It also decreases the formation of MDA and 8-oxodeoxyguanosine [64]. In a randomized, double-blind, placebo-controlled phase II clinical trial, short-term treatment with DFP in early-onset PD patients demonstrated safety and a reduction in iron levels in specific brain regions, although no significant therapeutic effect was observed [65]. Another pilot phase II trial demonstrated that DFP reduced SN iron deposition and slowed the progression of motor dysfunction in early-onset PD patients. This trial highlighted that a moderate DFP regimen can benefit patients without significantly interfering with physiological iron-dependent processes [64]. Postmortem analyses of PD patient brains revealed reduced transferrin (Tf) levels in the SN. The subcutaneous injection of Tf in MPTP-induced PD mouse models reduced iron accumulation in the brain and improved motor deficits. However, the non-targeted nature of Tf supplementation led to systemic iron depletion, making it less ideal for PD treatment. Nevertheless, this finding further supports iron chelation as a therapeutic strategy for PD [66].

To address the systemic side effects associated with traditional iron chelators, PBT434 has been developed. PBT434 is a novel quinazolinone compound with weak iron chelation properties. Studies have shown that PBT434 can inhibit iron-mediated redox activity and iron-mediated α-syn aggregation in vitro. In 6-OHDA and MPTP-induced PD mouse models, as well as in transgenic mouse models (*hA35T* α-syn), PBT434 can prevent the loss of neurons in the SN and reduce α-syn accumulation by lowering oxidative damage markers such as H_2_O_2_ and increasing the levels of FPN and DJ-1 [67]. Although the side effects of DFP can be reduced by carefully controlling the dosage, maintaining the appropriate dose is challenging and may impact therapeutic outcomes. PBT434, as a weaker iron chelator, offers the potential for higher dosages with better absorption, greater therapeutic effects, and reduced side effects compared to DFP.

#### 3.1.2. Iron Chelators with Multi-Dimensional Function

Beyond simple iron chelation, certain compounds exhibit additional therapeutic functions for PD, offering promising avenues for the development of multi-target PD drugs. For example, VK-28 and its derivatives not only possess iron chelation properties but also exhibit MAO inhibitory activity, which may help mitigate neurodegeneration. Clioquinol reduces intracellular iron levels while simultaneously inhibiting the AKT/mTOR pathway, potentially providing neuroprotection. Furthermore, certain flavonoid components demonstrate anti-inflammatory and antioxidant effects in addition to their iron-chelating capabilities. By simultaneously targeting multiple PD pathways while reducing intracellular iron levels, these compounds may enhance therapeutic outcomes. This multi-dimensional approach represents a promising direction for future PD drug development.

##### Hydroxyquinoline

**Figure 4:**
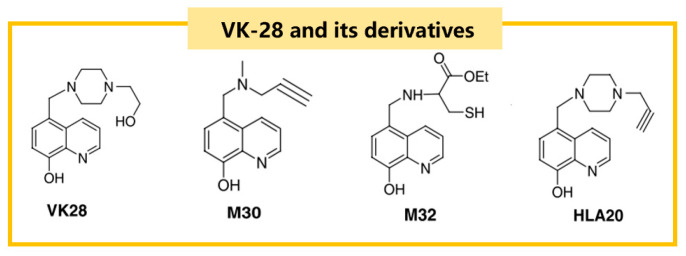
The structural formulas of VK-28 and its derivatives, including M30 and HLA20, which are notable for their iron-chelating properties and ability to cross the blood–brain barrier.

VK-28 and its derivatives are a class of iron chelators with a hydroxyquinoline skeleton that can cross the BBB. The structural formula of VK-28 and its derivatives are shown in Figure 4 [68]. VK-28 can inhibit iron/ascorbate-induced mitochondrial membrane lipid peroxidation. Pretreatment of rats with VK-28 induces neuroprotection against 6-OHDA neurotoxicity, preventing the reduction of dopaminergic neurons without affecting serotonin (5-HT) and norepinephrine metabolism [69].

Pretreatment with M30, a derivative of VK-28, protects the SN from lactacystin-induced dopaminergic neuron loss, iron accumulation, microglial activation, proteasome inhibition, and decreased bcl-2 levels. Additionally, M30 has a restorative effect on lactacystin-induced neurodegeneration [70]. HLA20, another derivative of VK-28, is an iron chelator with high free radical scavenging capacity and good membrane permeability to K562 cells, showing neuroprotective effects against 6-OHDA-induced differentiated P19 cell death [71]. Anti-ferroptosis tests of M30, HLA20, and M32 (another VK-28 derivative) demonstrated that these compounds could inhibit iron-dependent lipid peroxidation in rat brain homogenates, with effects comparable to DFO. In PC12 cells, they also mitigated cell death and neurotoxicity induced by 6-OHDA and serum deprivation [68].

Furthermore, it is worth noting that monoamine oxidase (MAO) is a naturally occurring enzyme in the human body that catalyzes the oxidation and deamination of monoamines and is divided into type A and type B. Type B is closely related to PD and participates in the inactivation of neurotransmitters such as dopamine in the brain. Existing studies have shown that MAO-B inhibitors can protect neurons by inhibiting α-synuclein aggregation, reducing oxidative stress, preventing apoptosis, and providing neurotrophic effects. HLA20 showed moderate selective MAO-B inhibitory activity [68]. M30 also exhibited highly effective non-selective MAO-A and MAO-B inhibition due to the propyl portion of the anti-PD drug Rasagiline [68]. The dual effect of iron chelators and MAO inhibition may help to improve the efficacy of Hydroxyquinoline in the treatment of PD.

##### Clioquinol

Clioquinol (CQ) is an iodinated 8-hydroxyquinoline (8-HQ), which has been widely used as an anti-parasitic agent for decades [72]. However, CQ also plays an important role in ferroptosis. In MPTP-induced monkey models, CQ acts as an iron chelator, and decreases iron content in the SN by up-regulating the expression of FPN1 and down-regulating the expression of the iron uptake transporter TFR2. CQ also decreases ROS content and 4-HNE levels while increasing GSH levels in the SN. Furthermore, CQ increases serum SOD and GSH levels, decreases serum MDA levels, promotes the AKT/mTOR pathway, and blocks p53-mediated cell death [73]. Therefore, CQ can regulate both intracellular iron content and AKT/mTOR pathway in the treatment of PD.

##### Flavonoids

Curcumin is a plant polyphenolic compound and a major component of the spice turmeric (*Curcuma longa*). As a member of the flavonoids, curcumin exhibits notable anti-inflammatory, antioxidant, and iron-chelating activities. In 6-OHDA-induced PD rat models, curcumin has been shown to reverse the decrease in dopamine content and TH immunoreactive neurons in the striatum, and reduce the number of iron-stained cells induced by 6-OHDA. Although the specific mechanism remains unclear, it may be related to curcumin’s iron chelation properties [74], indicating a potential role in anti-ferroptosis.

Baicalein (5,6,7-trihydroxy-2-phenyl-4H-1-benzopyran-4-one), a flavonoid derived from *Scutellaria baicalensis*, is prescribed for oxidative stress-related diseases [75]. In vitamin K-induced SK-N-MC cell models, baicalein has been shown to chelate free iron, reducing intracellular iron content and decreasing ROS and MDA levels. Baicalein also inhibits the activities of Bax and caspase-9 while inducing bcl-2 expression, preventing cell death [75]. In MPTP-induced PD mouse models, low-dose baicalein has demonstrated neuroprotective effects, improving motor ability and preventing the loss of dopaminergic neurons induced by MPTP. Studies indicate that baicalein reduces the activation of microglia and astrocytes by inhibiting the nuclear shift of NF-κB and reducing the activation of JNK and ERK, thereby achieving neuroprotective effects [76]. Additionally, in SN4741 cells, baicalein covalently binds to α-syn, potentially reducing α-syn transmission by promoting its polymerization into large complexes, and enhancing cell survival [77]. The iron chelating properties of flavonoids combined with their anti-inflammatory and antioxidant properties make them potentially a good dietary supplement to prevent PD from multiple angles.

#### 3.1.3. Other Iron Metabolism-Related Drugs

Apoferritin, an iron-free form of ferritin, has been widely used as a non-toxic nanomaterial in clinical applications such as drug delivery, in vivo imaging, and photothermal therapy. Interestingly, apoferritin also shows potential in inhibiting ferroptosis. In MPTP-induced PD mouse models, apoferritin downregulated the expression of DMT1 and ACSL4 while upregulating FSP1 expression, without affecting GPX4 levels, thereby preventing ferroptosis [78].

ACO1, a key enzyme that converts citrate to isocitrate, facilitates the synthesis of glutamate, contributing to ferroptosis resistance. However, elevated intracellular iron levels promote the conversion of IRP1 to ACO1, reflecting a paradox between iron-induced ferroptosis and ACO1-mediated ferroptosis inhibition. Berry et al. suggest that nerve damage in PD may result from dysregulated iron utilization. In rotenone-induced PD rat models, iron carbonyl administered via gavage strictly controlled iron utilization, reduced IRP1 activity, enhanced ACO1 activity, and ultimately prevented ferroptosis [79]. This approach highlights iron carbonyl as a novel strategy to mitigate ferroptosis in PD.

microRNAs (miRNAs) also play a crucial regulatory role in iron metabolism by influencing post-transcriptional modifications and mRNA stability. FTH1 stores intracellular iron, and silencing FTH1 increases LIP, promoting ferroptosis. In 6-OHDA-induced PD models, miR-335 was found to target FTH1, silencing its expression and thereby facilitating ferroptosis. Inhibiting endogenous miR-335 may offer a therapeutic avenue to counteract ferroptosis [80]. Additionally, miR-221 targets TFR2 in MPP⁺-induced SH-SY5Y cells, and silencing TFR2 reduces LIP, helping to combat ferroptosis [81]. The exploration of miRNA-based regulatory mechanisms provides a promising foundation for the design of targeted drugs to combat ferroptosis in PD.

**Table 1:** Iron metabolism-related drugs and their mechanisms.

Drugs	Inducer/Cell or Animal Models	Pharmacology	Mechanism	Reference
**DFO**	MPTP/mice	↓motor deficiency ↑TH positive neurons ↓iron-positive cells in SN and striatum	iron chelation ↓α-syn, DMT1, TFR	[61,64]
**DFP**	MPTP/mice	↑GSH ↓MDA ↓8-oxodeoxyguanosine ↑dopamine in striatum	iron chelation ↓unstable LIP in mitochondria	[64]
**Transferrin**	MPTP/mice	↓motor deficiency	iron chelation	[66]
**PBT434**	6-OHDA/mice MPTP/mice Transgenic mice(hA35T α-syn)	↓neurons loss in SN ↓oxidative damage markers	iron chelation ↓α-syn in SN ↑FPN1 ↑DJ-1	[67]
**VK-28**	6-OHDA/rats	↓neurotoxicity ↑dopaminergic neurons	iron chelation	[69]
**M30**	Lactacystin/mice	↓dopaminergic neurons loss	iron chelation ↓MAO-A, MAO-B non-selectively ↑bcl-2 levels ↓microglial activation ↓proteasome inhibition	[70]
**HLA20**	6-OHDA/P19	neuroprotection	iron chelation ↓MAO-B selectively	[68]
**CQ**	MPTP/monkeys	↓iron in SN ↓ROS ↓4-HNE ↑serum SOD ↑GSH↓MDA	Iron chelator ↑FPN1 ↓TFR2 ↑AKT/mTOR ↓p53-mediated cell death	[73]
**Curcumin**	6-OHDA/rats	↑dopamine in striatum ↑TH positive neurons in striatum ↓iron-stained cells	iron chelation	[74]
**Baicalein**	Vitamin K/SK-N-MC	↓iron content ↓ROS ↓MDA	iron chelation ↓Bax ↓caspase-9 ↑bcl-2	[75]
	MPTP/mice	↓motor deficiency ↑dopaminergic neurons	iron chelation ↓nuclear shift of NF-κB	[76]
	SN4741 cells	↓α-syn transmission	covalently bound to α-syn	[77]
**Apoferritin**	MPTP/mice	↓LIP	↓DMT1, ACSL4 ↑FSP1	[78]
**Iron carbonyl**	Retenone/rat	Control iron utilization	↓IRP1 ↑ACO1	[79]
**miR-335**	6-OHDA/rats 6-OHDA/PC12	↑LIP	↓FTH1	[80]
**miR-221**	MPP^+^/SH-SY5Y	↓LIP	↓TFR2	[81]

↑: up-regulate or increase; ↓: down-regulate or decrease

### 3.2. Decreasing LPO Generation

**Figure 5:**
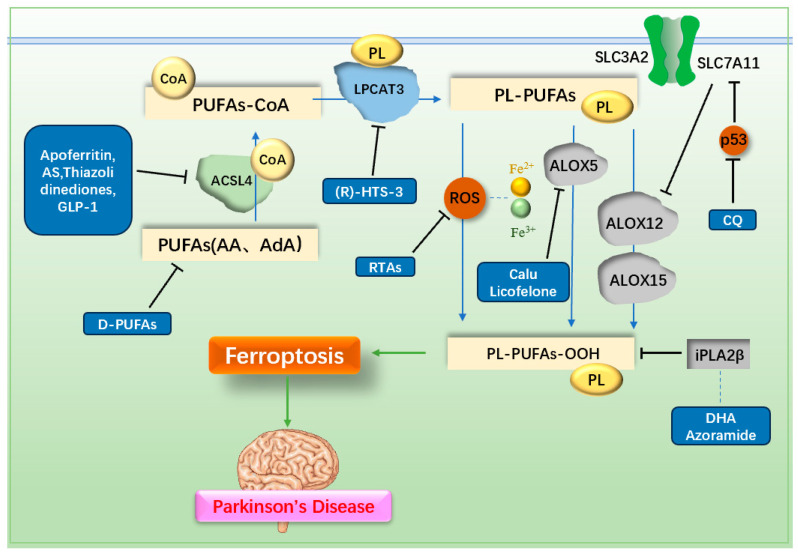
Mechanisms of lipid metabolism and related drugs. PUFAs are metabolized by ACSL4 and LPCAT3, then oxidized to LPO through enzymatic reactions mediated by ALOX or non-enzymatic reactions driven by ROS, leading to ferroptosis. Inhibiting PUFAs, ACSL4, LPCAT3, ROS, or ALOX reduces LPO production, thereby preventing ferroptosis. PUFAs: polyunsaturated fatty acids; D-PUFAs: tritiated unsaturated fatty acids; ACSL4: acyl CoA synthase long-chain member 4; LPCAT3: lysophosphatidylcholine acyltransferase 3; PL: phosphatidylethanolamine; ALOX: lipoxygenase; AS: AS-252424; GLP-1: glucagon-like peptide-1; RTAs: radical trapping antioxidants; Calu: clausenamide; CQ: clioquinol; DHA: docosahexaenoic acid.

A defining feature of ferroptosis is the accumulation of LPO. Consequently, directly inhibiting LPO production is a crucial anti-ferroptosis strategy. The generation of LPO is a complex process involving interactions among PUFAs, CoA, membrane phosphatidylethanolamine (PL), and various enzymes. PUFAs, especially arachidonic acid (AA) and adrenal acid (AdA), are the main substrates of LPO. Initially, PUFAs bind to CoA via the action of ACSL4, forming PUFA-CoA derivatives [61]. These PUFA-CoA derivatives are subsequently incorporated into membrane PL by lysophosphatidylcholine acyltransferase 3 (LPCAT3), producing PL-PUFAs [82]. PL-PUFAs are then oxidized to PL-PUFA-OOH via two main pathways. In the enzymatic pathway, Lipoxygenases (ALOXs), particularly ALOX12 and ALOX15, play a critical role in promoting PL-PUFA peroxidation [82]. Notably, ALOX12 is also implicated in p53-mediated ferroptosis [83]. ALOX5 has been reported to contribute to neuroinflammation in MPTP-induced PD mouse models [84]. In the non-enzymatic pathway, ROS generated via the Fenton and Haber–Weiss reactions drive the peroxidation of PL-PUFAs. Cytochrome P450 reductase and cytochrome b5 reductase 1 (CYB5R1) can further transfer electrons from NADPH to O_2_, producing H_2_O_2_ and exacerbating LPO through ROS-driven processes [85]. Given these mechanisms, targeting key enzymes involved in LPO production, such as ACSL4, LPCAT3, and ALOXs, or reducing the availability of PUFA substrates represents an important direction for anti-ferroptosis therapy. The metabolism of LPO and the mechanisms of drugs targeting ferroptosis in PD are illustrated in Figure 5 and listed in Table 2.

#### 3.2.1. Radical Trapping Antioxidants (RTAs)

Since the production of ROS is a key factor in LPO formation, RTAs can target and reduce ROS, playing a significant role in anti-ferroptosis therapy. Despite the two pathways of LPO production—enzymatic and non-enzymatic—RTAs have demonstrated efficacy in PD treatment. A randomized, double-blind, placebo-controlled phase II clinical trial showed that combining the endogenous antioxidant vitamin E with ω-3 fatty acids significantly improved total antioxidant capacity (TAC) and GSH levels in PD patients [86]. In RSL3-induced neuronal ferroptosis models, Diacetyl-bis(4-methyl-3-thiosemicarbazonato) copper(II) (Cu^II^(atsm)) effectively inhibited neuronal ferroptosis via its anti-LPO mechanism, which is attributed to its RTA activity [87]. In animal experiments, Cu^II^(atsm) improved disease progression in PD mouse models. Furthermore, a phase I clinical trial demonstrated that Cu^II^(atsm) significantly enhanced the cognitive abilities of PD patients [88]. This compound also exhibits good oral bioavailability and the ability to cross the BBB.

Advancements in RTA research have led to the discovery of compounds such as Fer-1 and Liproxstatin-1 [41], which have shown superior efficacy compared to vitamin E [89]. Fer-1, a classic RTA in ferroptosis studies, mitigates ferroptosis by increasing GPX4 expression through the Nrf2 pathway. It also reduces MDA concentration and increases superoxide dismutase (SOD) activity, thereby lowering intracellular ROS and LPO production [45]. When Fer-1 was injected into the brains of MPTP-induced acute PD mouse models, it significantly improved motor function. As an arylalkylamine, Fer-1 prevents membrane lipid damage due to its antioxidant properties. However, it suffers from low activity, poor stability, and low solubility. Through structure-activity relationship (SAR) studies, more potent Fer-1 analogues such as SRS11-92, SRS12-45, SRS13-35, and SRS13-37 have been synthesized, with SRS11-92 being 15 times more potent than Fer-1 [90]. The unstable ester component of Fer-1 leads to rapid hydrolysis into its inactive carboxylic acid form. Although SRS11-92 is highly effective compared to Fer-1, it does not resolve this instability issue. To address this, the unstable ester part of Fer-1 was replaced with a more stable sulfonamide group, resulting in the synthesis of UAMC-2418, which greatly improved stability and effectiveness [91].

**Figure 6:**
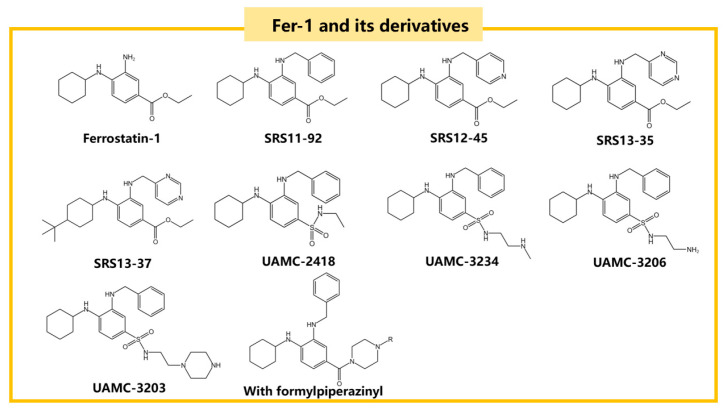
The structural formulas of Fer-1 and its derivatives, including SRS11-92, SRS12-45, and UAMC-2418, which are known for their anti-ferroptosis activity.

Further derivation of the aliphatic chain end on the sulfonamide part led to the synthesis of UAMC-3234, UAMC-3206, and UAMC-3203. These compounds were significantly superior to Fer-1 in protecting against acute iron poisoning in mice and showed excellent efficacy and therapeutic value in vivo [91]. Additionally, introducing a formylpiperazine group to Fer-1 enhances anti-iron deposition activity and solubility. Compounds containing a 4-(mesulfonyl)benzyl group in the formylpiperazine part exhibit better anti-ferroptosis activity and microsomal stability than Fer-1 [92]. The structural formulas of Fer-1 and its derivatives are shown in Figure 6 [90,91,92].

Although modification based on SAR studies can enhance Fer-1’s efficacy as an anti-ferroptosis drug and reduce its side effects, Fer-1 cannot cross the BBB, severely limiting its application [45]. Whether modifying Fer-1 can enable it to overcome the BBB remains uncertain. Some researchers have synthesized CCM Fer-1 conjugates, which achieve the stable release of Fer-1. It is anticipated that these conjugates could facilitate Fer-1’s passage through the BBB [93]. Additionally, intranasal administration of nanoparticle drugs [94], intravenous administration of recombinant adeno-associated virus (AAV) carriers [95] and the use of exosomes [96]. are promising new methods for breaking though the BBB. The application of these delivery systems with Fer-1 and its derivatives is expected to enhance the therapeutic potential of Fer-1 in treating PD.

2,2,6,6-Tetramethylpiperidine-N-Oxyl (TEMPO) is a distinctive RTA with the ability to act remotely. It exhibits high stability, can be volatilized into the air in a stable form, and is easily dissolved in liquids. In glutamate-induced mouse hippocampal cell lines, TEMPO has been shown to inhibit cell death, highlighting its potential as a therapeutic RTA for the inhaled treatment of PD [89]. In addition to TEMPO, other RTA drugs, such as aromatic amine RTAs, effectively inhibit LPO production through their RTA activity. However, further research is needed to identify an RTA with high potency and minimal side effects suitable for clinical application. Current advancements in RTA development suggest that this goal is within reach.

#### 3.2.2. ACSL4 Inhibitor

ACSL4 is an enzyme critical for LPO production and can promote ferroptosis by converting PUFAs to acyl-CoA, especially adrenic acid (C22:4) and arachidonic acid (C20:4) [97,98,99]. Further research has identified AS-252424 (AS), a substance that specifically inhibits ACSL4. In ferroptosis models, including RSL-induced MDA-MB-231, Hela, A549, L-02, HK-2, HT22, MCA205, and B16-F10 cells, as well as HT1080 cells induced by ferroptosis inducers erastin and FIN56, AS demonstrated a strong inhibitory effect on ferroptosis [100]. However, AS faces challenges such as poor solubility, short half-life, and high clearance rate. To address these issues, researchers developed a methoxy poly(ethylene glycol)-poly (ε-caprolactone) (mPEG-PCL) copolymer-based nanoparticle for AS delivery, which yielded promising results [100]. Glucagon-like peptide-1 (GLP-1), a drug commonly used in treating diabetes and obesity, has also been shown to have ACSL4 inhibitory activity in MPTP-induced mice models [101].

#### 3.2.3. LPCAT3 Inhibitor

Reed et al. identified (R)-HTS-3 as a stereoselective inhibitor of LPCAT3 and demonstrated its inhibitory effect on ferroptosis. However, its role in PD models remains unverified. Furthermore, their findings suggest that the loss of ACSL4 provides greater protection against ferroptosis compared to LPCAT3 inhibition [102]. This indicates that the therapeutic potential of developing LPCAT3 inhibitors may be limited.

#### 3.2.4. ALOX5 Inhibitor

Although the inhibition of lipoxygenases (ALOXs) may seem less effective due to the presence of multiple subtypes and pathways contributing to LPO production, drugs targeting ALOX5 have shown promising results in ferroptosis inhibition. Clausenamide (calu), a natural racemic pyrrolidone compound isolated from the leaves of *Clausena lansium* (Lour.), has been shown to inhibit LPO in the brains of MPTP-induced PD mouse models by inhibiting ALOX5 activation and nuclear translocation, thereby preventing ferroptosis of dopaminergic neurons [103]. Licofelone, an osteoarthritis drug approved for its dual inhibition of cyclooxygenase (COX) and ALOX5 pathways, also demonstrates protective effects in PD. In MPTP-induced PD mouse models, licofelone improves locomotor ability and reduces oxidative damage [104]. Among the various ALOX subtypes, ALOX5 appears to play a central role in LPO generation. This may explain why the inhibition of ALOX5 yields more pronounced effects in reducing ferroptosis compared to other ALOX subtypes.

#### 3.2.5. PUFAs Inhibitor

PUFAs are essential substrates for LPO, making their inhibition a promising approach to reducing LPO formation. Tritiated unsaturated fatty acids (D-PUFAs) can compete with PUFAs to prevent PUFA oxidation, and supplementation with D-PUFAs can help gradually reshape cell membranes and prevent LPO [105]. Studies have shown that cells pre-cultured with D-PUFAs can prevent α-syn-induced LPO and α-syn-induced cell death [106]. Therefore, the application of D-PUFAs can essentially inhibit LPO to a certain extent, exerting an anti-ferroptosis effect. However, the use of D-PUFAs for anti-ferroptosis carries significant risks. While they are effective at preventing LPO, tritium atoms are radioactive and undergo beta decay to produce beta particles. Injecting D-PUFAs into the human body may cause internal exposure, leading to potential carcinogenicity or other injuries. Therefore, the use of D-PUFAs as an anti-ferroptosis agent requires extensive research to verify their safety and efficacy.

#### 3.2.6. iPLA2β-Related Drugs

Calcium-independent phospholipase A2β (iPLA2β), encoded by the *PLA2G6* gene, is a critical enzyme in the calcium-dependent phospholipase A2 family. Mutations in the *PLA2G6* gene are associated with young-onset dystonia-parkinsonism type 14 (*PARK14*) [107]. iPLA2β plays a key role in lipid regulation linked to ferroptosis. One mechanism of ferroptosis involves ALOX12, an enzyme required for LPO production. Normally, the direct binding of SLC7A11 to ALOX12 inhibits its activity. However, p53 suppresses SLC7A11, indirectly promoting ALOX12 activity and driving ferroptosis. iPLA2β has been shown to inhibit this p53-driven ferroptosis through a GPX4-independent mechanism, although the exact details remain unclear [83,108]. Dietary supplementation with docosahexaenoic acid (DHA), a product of iPLA2β activation, has been shown to alleviate motor deficits in *PLA2G6^D331Y/D331Y^* mice by reducing neuroinflammation and lipid peroxidation [109]. Additionally, azoramide, a compound that restores ER function and activates cAMP-response element binding protein (CREB) signaling, can mitigate dopaminergic neurons damage caused by *PLA2G6* mutations [107].

Interestingly, while iPLA2β plays an anti-ferroptosis role in lipid regulation, it exhibits a paradoxical role in iron metabolism. In SH-SY5Y cells, iPLA2β decreases ferritin expression and increases TFR1 expression without altering FPN1 or DMT1 levels, leading to increased iron uptake and reduced iron storage [110]. This suggests that iPLA2β can promote elevated LIP levels and ferroptosis. As a result, further studies are needed to reassess the viability of iPLA2β as a therapeutic target for anti-ferroptosis treatment in PD.

**Table 2:** LPO metabolism-related drugs and their mechanisms.

Drugs	Inducer/Cell or Animal Models	Pharmacology	Mechanism	Reference
**Cu** **^II^** **(astm)**	RSL3/mice cortical neurons	↓LPO	RTA	[87]
**Fer-1**	Glutamate/HT-22	↑GPX4 ↓MDA ↑SOD ↓ROS ↓LPO	RTA ↑Nrf2/GPX4	[45]
**TEMPO**	Glutamate/mouse hippocampal cell lines	↓LPO ↓cell death	RTA	[89]
**AS**	RSL3/HT22	↓LPO	↓ACSL4	[100]
**Calusenamide**	MPTP/mice	↓LPO	↓ALOX5 activation/nuclear translocation	[103]
**Licofelone**	MPTP/mice	↑locomotor ability	↓ALOX5	[104]
**D-PUFAs**	Mixed cultures of cortical neurons of rats	↓α-syn-induced LPO and α-syn-induced cell death	compete with PUFAs	[105,106]
**DHA**	*PLA2G6^D331Y/D331Y^* mice	Improve motor deficits ↓neuroinflammation	↓LPO	[109]
**Azoramide**	*PLA2G6^D331Y^* dopaminergic neurons	Improve DA damage	↑ER function ↑CREB signaling	[107]

↑: up-regulate or increase; ↓: down-regulate or decrease.

### 3.3. Regulating Antioxidant Pathway

**Figure 7:**
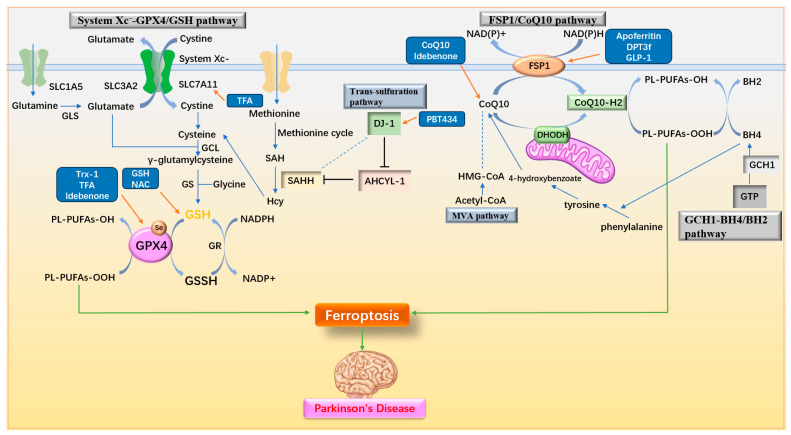
Antioxidant pathways and drug mechanisms. (1) The System Xc^−^-GPX4/GSH pathway facilitates the intracellular transport of cystine, which is converted to cysteine for GSH synthesis, providing substrates for the antioxidant activity of GPX4. (2) The FSP1/CoQ_10_ pathway converts CoQ_10_ to its reduced form, CoQ_10_-H2, through the actions of FSP1 and DHODH, exerting antioxidant effects. (3) The GCH1-BH4/BH2 pathway produces BH4 via GCH1, enhancing cellular antioxidant capacity and contributing to CoQ_10_ synthesis. (4) In the trans-sulfuration pathway, DJ-1 indirectly promotes SAHH activity, increasing Hcy and cysteine production. (5) The MVA pathway supports CoQ10 biosynthesis. Upregulation of System Xc^−^, GPX4, GSH, FSP1, CoQ10, DJ-1, and related factors effectively inhibits ferroptosis. GSH: glutathione; GLS: Glutaminases; GCL: γ-glutamylcysteine ligase; GS: GSH synthetase; GR: glutathione reductase; GPX4: Glutathione Peroxidase 4; SAH: S-adenosyl homocysteine; SAHH: S-adenosyl homocysteine hydrolase; Hcy: homocysteine; AHCYL-1: adenosyl homocysteinase like 1; FSP1: ferroptosis inhibitory protein 1; DHODH: dihydroorotate dehydrogenase; GCH1: GTP cyclohydrolase; BH4/BH2: tetrahydrobiopterin/dihydrobiopterin; MVA pathway: mevalonate pathway; Trx-1: Thioredoxin-1; TFA: Total flavonoids of Astragalus membranaceus; NAC: N-acetylcysteine; GLP-1: glucagon-like peptide-1.

The antioxidant system plays a crucial role in cellular resistance to ferroptosis. Many drugs have been identified that alleviate or treat PD by stimulating various antioxidant pathways to counteract ferroptosis. These pathways include mechanisms such as enhancing glutathione (GSH) synthesis, activating key enzymes like GPX4, and modulating additional antioxidant systems. The antioxidant pathways and associated drug mechanisms targeting ferroptosis in PD are illustrated in Figure 7 and summarized in Table 3.

#### 3.3.1. Regulating System Xc^−^-GPX4/GSH Pathway

The System Xc^−^-GPX4/GSH pathway plays a critical role in defending against ferroptosis. Enhancing GSH levels is particularly significant for anti-ferroptosis treatment in PD. In a double-blind, placebo-controlled phase II trial, intranasal administration of GSH (100 or 200 mg once daily for three months) improved PD symptoms, but the results were not significantly different from the placebo [111]. However, in another phase II clinical trial using a higher dose of GSH (600 mg twice daily for 30 days) administered intravenously, GSH demonstrated an effect in improving PD symptoms and potentially delaying disease progression [112]. As a precursor to cysteine, NAC is a classic antioxidant. In an open-label prospective study, oral NAC (6000 mg daily for 28 days) increased peripheral antioxidant capacity but failed to raise brain GSH levels, likely due to its low bioavailability [113]. Conversely, in a phase II clinical trial using intravenous NAC at a similar dose (150 mg/kg), increased brain GSH levels were observed, indicating its potential for treating PD [114]. These findings suggest that appropriate doses of GSH or NAC, combined with more effective delivery methods (e.g., intravenous injection), may serve as viable therapies for PD, although further clinical trials are needed to confirm efficacy and safety.

In addition, promoting GPX4 or System Xc^−^ activity is also important. Selenium is an essential element for maintaining GPX4’s catalytic function [115]. While selenium supplementation has been proposed as a method to prevent ferroptosis, a case-control study indicated that selenium reduction in PD patients is not significant, suggesting its therapeutic value may be limited [116]. Additionally, thioredoxin-1 (Trx-1) has been shown to reverse GPX4 decline in PC12 and SH-SY5Y cells exposed to MPP^+^, as well as in the SN of MPTP-induced PD mouse models [117]. This indicates that Trx-1 can regulate GPX4 expression, offering another potential avenue for ferroptosis prevention.

Some flavonoids, such as curcumin and baicalein, exhibit anti-ferroptosis properties due to their iron-chelating activity. Total flavonoids of Astragalus membranaceus (TFA), derived from Astragalus membranaceus extract, play a significant role in regulating the System Xc^−^-GPX4 pathway. In MPTP-induced PD mouse models, TFA administration via gavage increased the expression of SLC7A11 and GPX4, raised the GSH/GSSG ratio, enhanced cellular antioxidant capacity, and inhibited neuronal ferroptosis [118]. In a randomized, double-blind, placebo-controlled feasibility study, daily cocoa containing flavonoids showed some ability to alleviate fatigue symptoms in PD patients, though not significantly [119]. Another double-blind, placebo-controlled, parallel-group study demonstrated that multiple doses of a flavonoid drug (200–800 mg daily) exhibited good safety and tolerability without serious accumulation, further supporting the potential of flavonoid-based therapies as dietary supplements for PD [120].

#### 3.3.2. Regulating Nrf2-Related Pathway

Nuclear factor erythroid-2-related factor 2 (Nrf2) is a key regulator in cellular antioxidant processes. It exerts its effects by binding to antioxidant response elements (ARE) to promote the transcription of antioxidant genes. However, the intracellular half-life of Nrf2 is limited to 10–15 min due to the regulatory role of Kelch-like ECH-associated protein 1 (Keap1). Keap1 forms a dimer with CULLIN3 (CUL3), the scaffold protein of the E3 ubiquitin ligase complex, to ubiquitinate and degrade Nrf2 via the 26S proteasome under normal physiological conditions [121]. However, under oxidative stress, Keap1-mediated Nrf2 degradation is inhibited, allowing Nrf2 to translocate into the nucleus and bind to ARE, thereby regulating downstream antioxidant gene transcription [122]. Additionally, DJ-1, a regulatory factor in the trans-sulfuration pathway, promotes the dissociation of the Keap1/Nrf2 complex, enhancing Nrf2 expression and downstream gene activity. This mechanism makes DJ-1 a potential target for certain drugs [123].

Nrf2 plays a critical role in regulating factors associated with ferroptosis. It controls the expression of iron metabolism proteins, such as ferritin and FPN [124], as well as key components of the System Xc^−^-GPX4/GSH pathway, including GCL, GS, GPX4 [125], and SLC7A11 [126]. Furthermore, Nrf2 regulates CBS in the trans-sulfuration pathway to increase intracellular cysteine levels. Nrf2 also influences the expression of NAD(P)H:quinone oxidoreductase 1 (NQO1) [127] and heme oxygenase-1 (HO-1) [128], both of which enhance cellular antioxidant capacity. Given its central role, Nrf2 is an important target for developing anti-ferroptosis therapies for PD.

**Figure 8:**
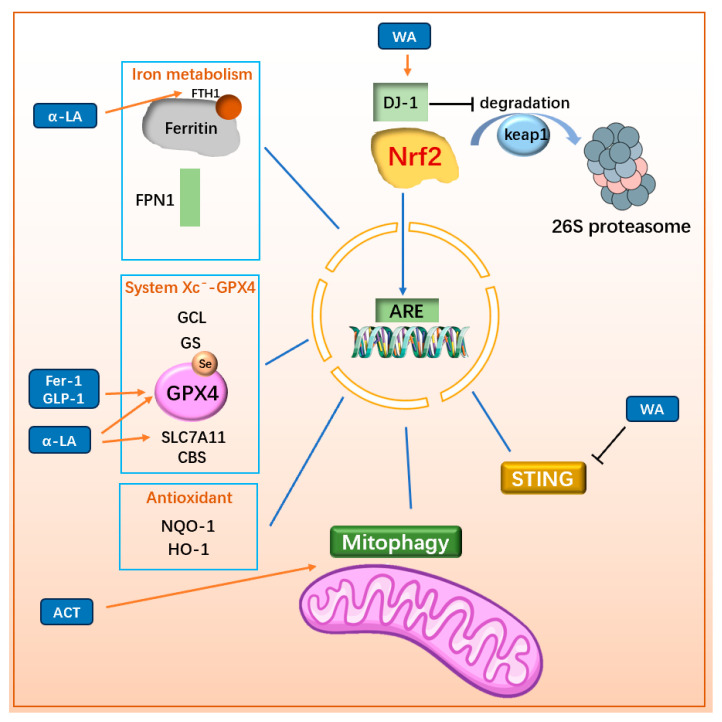
Drugs inhibit ferroptosis through Nrf2-related mechanisms. Nrf2 binds to AREs in the promoters of antioxidant genes, regulating the expression of ferroptosis-related genes such as ferritin, FPN1, GCL, GS, GPX4, SLC7A11, CBS, NQO1, and HO-1, thereby enhancing the cell’s antioxidant capacity. Nrf2 also influences pathways controlling mitophagy and suppressing STING-mediated neuroinflammation. The degradation of Nrf2 occurs through the Keap1-mediated 26S proteasome pathway, but DJ-1 inhibits this degradation, thereby promoting Nrf2 stability and function. The upregulation of Nrf2 enhances antioxidant defenses, promotes mitophagy, and mitigates neuroinflammation. Similarly, enhancing DJ-1 function further supports Nrf2 activation and its protective effects. FTH1: ferritin H chain; FPN1: ferroportin 1; GCL: γ-glutamylcysteine ligase; GS: GSH synthetase; GPX4: Glutathione Peroxidase 4; CBS: cystathionine-β-synthase; NQO1: NAD(P)H dehydrogenase quinone; 1Nrf2: Nuclear factor erythroid-2-related factor 2; Keap1: Kelch-like ECH-associated protein 1; ARE: antioxidant response elements; α-LA: α-Lipoic acid; Fer-1: ferrostatin-1; GLP-1: glucagon-like peptide-1; ACT: Acteoside; WA: Withaferin A; STING: stimulator of interferon genes.

**Figure 9:**
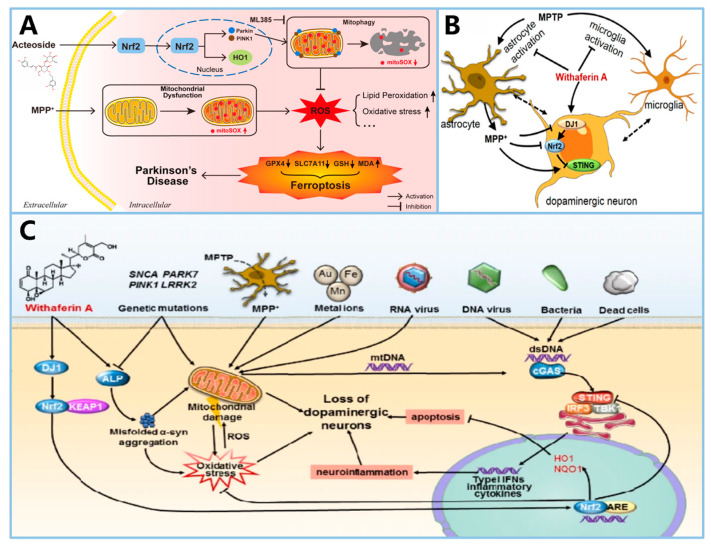
Summary of the mechanisms of action of Acteoside and Withaferin A in PD. (**A**) shows the mechanism by which ACT inhibits ferroptosis in PD neurons by enhancing mitophagy and reducing lipid peroxidation via the Nrf2 pathway (reprinted with permission from Ref. [132] Copyright 2024, ELSEVIER). (**B**,**C**) illustrate the neuroprotective actions of WA in PD, highlighting its modulation of the DJ1-Nrf2-STING pathway and its impact on microglia activation and dopaminergic neuron survival (reprinted with permission from Ref. [133] Copyright 2021, Springer Nature).

For example, a naturally synthesized small molecule, α-Lipoic acid (α-LA) can alleviate MPP⁺-induced ferroptosis in PC12 cells by activating the PI3K/Akt/Nrf2 pathway. This activation decreases levels of MDA, 4-HNE, iron, and ROS, while increasing the levels of SLC7A11 and GPX4, thereby inhibiting ferroptosis and exerting a protective effect on cells [129]. In 6-OHDA-induced PC12 cell models, α-LA rescues mitochondrial damage, decreases intracellular iron and LPO content, and improves ferroptosis by activating the SIRT1/Nrf2 pathway, upregulating ferritin heavy chain 1 (FTH1) and GPX4 levels [130]. Known for its RTA activity, Fer-1 plays a vital role in ferroptosis resistance. It also promotes the Nrf2/GPX4 pathway, further strengthening its anti-ferroptosis effects [45]. In addition, Nrf2 also plays a role in promoting mitophagy. For example, Acteoside (ACT), a primary active component derived from the Scrophulariaceae family plant Cistanche, can promote mitophagy primarily by enhancing the PINK1/Parkin pathway through the Nrf2-mitophagy axis [131], thereby improving mitochondrial integrity in dopaminergic neurons and reducing LPO [132]. Since DJ1 can enhance Nrf2 content by promoting the dissociation of Keap1/Nrf2, withaferin A (WA) has been shown to reduce the loss of dopaminergic neurons, decrease neuroinflammation, and alleviate motor deficits in MPTP-induced mouse models by targeting the DJ1-Nrf2-STING pathway in dopaminergic neurons [133]. Within the DJ1-Nrf2-STING pathway, STING (stimulator of interferon genes, TMEM173) is known as an innate immune response regulator that induces type-I interferon (IFN) signaling and participates in neuronal immunomodulation [134,135]. Activation of STING can accelerate the formation of Lewy bodies and contribute to the loss of dopaminergic neurons [136,137]. WA activates DJ1 in dopaminergic neurons, thereby reducing STING-mediated neuroinflammation through the DJ1-Nrf2 axis and enhancing the anti-oxidative stress capacity of DJ1 in these neurons. These findings underscore the therapeutic potential of Nrf2 in regulating ferroptosis and addressing neuroinflammation in PD. The mechanisms and drugs targeting Nrf2 are illustrated in Figure 8 and Figure 9.

#### 3.3.3. Regulating FSP1/CoQ_10_ Pathway

The FSP1/CoQ_10_ pathway plays a key role in producing CoQ_10_ and mediating antioxidant effects, making CoQ_10_ supplementation a potential therapeutic strategy for treating PD by counteracting ferroptosis.

Direct dietary supplementation with CoQ_10_ has been shown to protect dopaminergic neurons and TH-positive neurons in the SN and reduce α-synuclein aggregation in MPTP-induced PD mouse models, resulting in neuroprotective effects [138]. In a phase III clinical trial involving a single dose of CoQ_10_ (100 mg three times daily for three weeks), no significant changes in PD symptoms were observed between the treatment and placebo groups, although CoQ_10_ was demonstrated to be safe and well-tolerated at this dose [139]. In contrast, a phase II clinical trial by Thomas et al. reported significant improvements in PD symptoms in patients receiving CoQ_10_ supplements (360 mg once daily for four weeks) compared to the placebo group [140].

**Figure 10:**
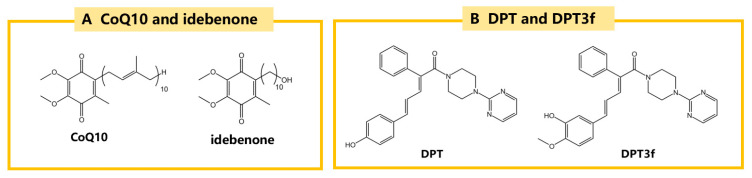
The structural formulas of CoQ_10_, idebenone and DPT and its derivative DPT3f. (**A**) CoQ_10_ and idebenone, potent antioxidants that protect against lipid peroxidation and oxidative stress. (**B**) DPT and its derivative DPT3f, which uniquely target the FSP1/CoQ_10_ pathway.

Given the challenges of CoQ10 crossing the BBB, idebenone has emerged as a promising analogue with better fat solubility. The hydroxydecyl side chain of idebenone is of an ideal length for favoring its partitioning into the mitochondrial membrane and enhancing BBB permeation compared to CoQ_10_ [141]. Idebenone has been shown to improve movement disorder symptoms in rotenone-induced PD rat models. The structural differences between CoQ_10_ and idebenone are shown in Figure 10A [142]. Idebenone has also been demonstrated to inhibit ferroptosis by increasing GPX4 expression. Furthermore, idebenone can prevent the decline in the expression of NQO1, an essential enzyme for mitochondrial respiration. This enhances the survival of TH positive neurons and provides additional protection against rotenone-induced damage [46]. Additionally, idebenone has been found to promote Parkin/PINK1 mitophagy in MPTP-induced mouse PD models, thereby clearing damaged mitochondria and improving PD symptoms [143].

In addition, a novel ferroptosis inhibitor, DPT was discovered through phenotypic assays in recent years. Among its various derivatives, DPT3f has demonstrated the most effective anti-ferroptosis activity due to its unique structure. The structures of DPT and DPT3f are shown in Figure 10B. Unlike conventional ferroptosis inhibitors that target the GPX4/GSH pathway, ACSL4, or reduce iron load, DPT3f acts primarily through the FSP1/CoQ_10_ pathway. In rat models of ischemic stroke, DPT3f has been shown to enhance the antioxidant activity of the FSP1/CoQ_10_ pathway by increasing FSP1 protein levels, thereby exhibiting significant anti-ferroptosis properties [144].

Statins, a widely used class of lipid-lowering drugs, also influence the production of endogenous CoQ_10_. HMG-CoA reductase, the key rate-limiting enzyme in the MVA pathway, is inhibited by statins, leading to reduced production of endogenous CoQ_10_. This reduction can sensitize cells to FIN56-induced ferroptosis and potentially promote the development of PD to some extent [37]. However, epidemiological studies suggest that statin use is associated with a reduced incidence of PD. This protective effect is likely related to the decreased risk of cerebrovascular atherosclerosis provided by statins [145]. These findings indicate that the inhibition of endogenous CoQ_10_ production by statins does not play a major role in PD development. On balance, the use of statins appears to be beneficial for preventing PD, despite their potential to promote ferroptosis by reducing CoQ_10_ levels.

#### 3.3.4. Multifunctional Antioxidant Regulator

In recent years, GLP-1 has gained attention not only for its role in treating diabetes and obesity but also for its neurotrophic effects. Studies have shown that GLP-1 has beneficial effects on PD patients [146]. However, the specific mechanisms remain unclear, and the short half-life of GLP-1 limits its use for long-term treatment of PD patients [147]. To address this, researchers developed a novel probiotic, *lactococcus lactis* MG1363-pMG36e-GLP-1, which can consistently express GLP-1. In MPTP-induced PD mouse models, this probiotic demonstrated neurotrophic effects. It was shown to down-regulate ACSL4, a key protein in LPO production, by activating the Keap1/Nrf2/GPX4 pathway and up-regulating FSP1, thereby exhibiting anti-ferroptosis properties. This probiotic also improved the survival of TH positive neurons in the SN and striatum, reduced α-syn deposition, and may enhance BBB integrity [101].

Furthermore, through the microbiota-gut-brain axis, the gut microbiome can affect not only gastrointestinal physiology but also central nervous system (CNS) function [148]. The gastrointestinal microbiome (GM) plays an increasingly prominent role in the pathogenesis and treatment of PD [149]. As a type of lactic acid bacteria, *lactococcus lactis* MG1363-pMG36e-GLP-1 has been shown to enhance the intestinal barrier and reverse dysbiosis. It may also play a therapeutic role in PD through the microbiota-gut-brain axis, although its specific mechanism needs further study [101]. Therefore, *lactococcus lactis* MG1363-pMG36e-GLP-1 represents a promising treatment for PD with multi-dimensional anti-ferroptosis action.

**Table 3:** Antioxidant-related drugs and their mechanisms.

Drugs	Inducer/Cell or Animal Models	Pharmacology	Mechanism	Reference
**Trx-1**	MPP^+^/PC12 MPP^+^/SH-SY5Y MPTP/mice	↑GSH	↑GPX4	[117]
**TFA**	MPTP/mice	↑GSH	↑SLC7A11/GPX4	[118]
**α-LA**	MPP^+^/PC12	↓MDA, 4-HNE, iron, ROS	↑PI3K/Akr/Nrf2/ SLC7A11/GPX4	[129]
	6-OHDA/PC12	↓iron, LPO ↓mitochondrial damage	↑SIRT1/Nrf2/ FTH1/GPX4	[130]
**Fer-1**	Glutamate/HT-22	↓MDA ↑SOD ↓ROS ↓LPO	RTA ↑Nrf2/GPX4	[45]
**ACT**	MPP^+^/SH-SY5Y MPTP/mice	↑mitochondrial integrity in dopaminergic neurons ↓LPO	↑Nrf2-mitophagy	[132]
**WA**	MPTP/mice	↓loss of dopaminergic neurons ↓neuroinflammation ↓movement disorder	↑DJ1/Nrf2 while ↓STING	[35,133]
**CoQ_10_**	MPTP/mice	↑survival of dopaminergic neurons and TH positive neurons ↓α-syn aggregation	↑CoQ_10_	[138]
**Idebenone**	rotenone/rats	↓movement disorder ↑survival of TH positive neurons	↑GPX4 ↑NAD(P)H dihydrogenase [quinone]-1	[46]
	MPTP/mice	improve PD symptoms	↑Parkin/PINK1 mitophagy	[143]
**DPT3f**	Ischemic stroke	↑CoQ_10_	↑FSP1/CoQ_10_	[144]
**Novel Probiotic *L. lactis* MG1363-pMG36e-GLP-1**	MPTP/mice	Neurotrophy ↑CoQ_10_ ↓LPO ↓α-syn ↑TH positive nerve cells ↑BBB integrity ↑intestinal barrier reverse dysbacteriosis	↑Keap1/Nrf2/GPX4 ↑FSP1/CoQ_10_ ↓ACSL4	[101]

↑: up-regulate or increase; ↓: down-regulate or decrease.

## 4. Conclusions

The pathogenesis of PD encompasses multiple factors, including α-syn accumulation, oxidative stress, mitochondrial dysfunction, microglia-associated neuroinflammation, and NM accumulation. Ferroptosis-related mechanisms, such as iron deposition, ROS overproduction, iron-dependent LPO and impaired LPO clearance, observed in the SN and dopaminergic neurons of PD patients, are closely linked to these pathogenic processes. These characteristics have guided the development of many drugs targeting ferroptosis to treat PD through these pathways.

Regulating iron homeostasis in dopaminergic neurons is a cornerstone of anti-ferroptosis strategies for PD treatment. Studies using iron chelators such as DFO, DFP, and Tf in PD mouse models have highlighted the therapeutic potential of iron chelation. Furthermore, multi-functional drugs like M30, CQ, and flavonoids, which combine iron chelation with other anti-PD effects, show promise for enhancing the efficacy of PD treatments through multi-dimensional approaches.

In addition to iron regulation, drugs that counteract ROS production and LPO formation caused by iron homeostasis dysregulation have demonstrated significant therapeutic effects. Strengthening intrinsic anti-ferroptosis and anti-oxidative stress pathways, such as those involving DJ-1, Nrf2, and GPX4, holds great potential. Emerging targets such as apoferritin, iPLA2β and IRPs are also being explored, offering new avenues for drug development against PD.

The potential role of the gut microbiome in neuroprotection is another intriguing area of research. For instance, *lactococcus lactis* MG1363-pMG36e-GLP-1 has shown neuroprotective effects, suggesting that the gut microbiome may contribute to anti-ferroptosis pathways. Studies on lysophosphatidylcholine (LPC), a gut microbiome-derived molecule, indicate its ability to improve AD pathology by inhibiting ACSL4 [150], hinting at its potential for neuroprotection in PD. However, the mechanisms underlying these effects remain unclear and warrant further investigation.

## 5. Future Perspectives

The complex and multifaceted pathogenesis of PD, combined with significant individual variability among patients, poses a challenge to the predictability of clinical trial outcomes. In addition, the current gold standard of ferroptosis is not clear, and researchers can only determine whether ferroptosis occurs through several key indicators, which also leads to a high degree of individual differences in clinical trials. This variability, however, also highlights opportunities for personalized treatment strategies targeting ferroptosis. Differences in iron metabolism and genetic profiles among patients suggest that tailoring anti-ferroptosis treatments to individual needs could maximize therapeutic benefits. For example, drugs with varying iron-chelation properties, such as the strong chelator DFO or the weaker chelator PBT434, could be selected based on a patient’s specific metabolic profile. Similarly, dosage optimization, as seen with GSH and NAC, could significantly affect treatment efficacy. The biochemical profiling of ferroptosis-related pathway activity, such as the System Xc^−^-GPX4/GSH or FSP1/CoQ_10_ pathways, could further refine drug selection. For instance, patients with impaired System Xc^−^-GPX4/GSH activity may benefit from GSH or NAC supplementation, while those with reduced FSP1/CoQ_10_ activity might respond better to idebenone.

Genetic differences also hold promise for precision medicine. For example, patients with *PARK7* mutations affecting DJ-1 may respond to DJ1-enhancing drugs like WA or PBT434, while those with *PARK14* mutations involving *PLA2G6* may benefit from DHA or azoramide. Although the pathological mechanism of PD is not clear, oxidative stress and mitochondrial damage are almost common in PD patients at present. Therefore, the personalized drugs above can be combined with GSH, NAC, and other anti-oxidative stress drugs to achieve better effects. However, implementing these strategies will require extensive clinical data to identify the most effective drug types, strengths, and dosages for each patient subgroup.

**Figure 11:**
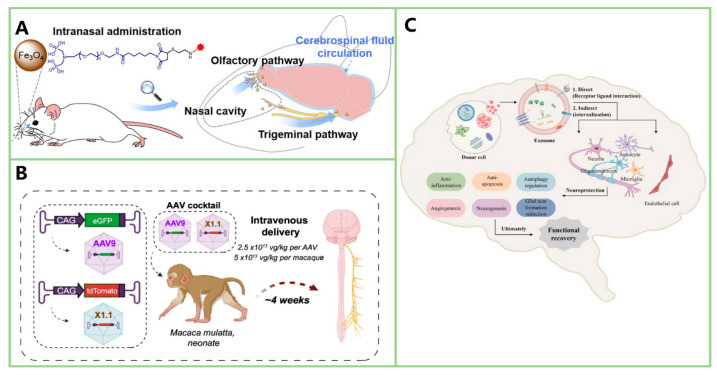
Illustration of three methods for crossing the BBB. (**A**) Intranasal administration of nanoparticles enables direct entry into the central nervous system via the olfactory and trigeminal pathways, bypassing the BBB. (Reprinted with permission from Ref. [94]. Copyright 2023, ACS). (**B**) Intravenous delivery of engineered adeno-associated viruses facilitates CNS transduction in rhesus macaques, showing potential for targeted gene therapy. (Reprinted with permission from Ref. [95]. Copyright 2022, Nature). (**C**) Exosomes, which can cross the BBB, mediate neuroprotective effects through various mechanisms, including angiogenesis, neurogenesis, and anti-apoptotic actions, contributing to functional recovery in neurological disorders. (Reprinted with permission from Ref. [96]. Copyright 2025, Wolters Kluwer Medknow).

Overcoming the BBB remains a significant obstacle in ferroptosis-targeted therapies. Recent advances in drug delivery methods, such as intranasal administration of nanoparticle-based drugs [94], intravenous administration of AAV carriers [95], and exosome-based delivery [96], offer promising solutions. The illustration of the three methods are shown in Figure 11. Intranasal administration provides a convenient route with significant brain penetration, though verifying drug delivery to target brain regions remains a challenge. Another disadvantage of this method is that it is easy to enter the lungs and cause damage such as aspiration pneumonia. At the same time, the clear function of mucus and cilia on respiratory mucosa also greatly affects the efficiency of nasal delivery of nanoparticle drugs. Intravenous AAV carriers allow precise dosage control and customizable targeting but require further development to simplify the identification of surface targets. Exosomes, with their low immunogenicity, good stability, and high delivery efficiency, offer another promising option, though the lack of standardized isolation and characterization methods currently limits their clinical application. Combining these delivery methods with existing anti-ferroptosis drugs, such as Fer-1 or its derivatives, is expected to enhance their therapeutic efficacy by improving BBB penetration.

Improving drug specificity for the SN is equally important to minimize systemic side effects. For instance, conjugating nanoparticles with BBB-penetrating peptides like RVG29 has shown promise in enhancing targeting efficiency [151]. Identifying specific targets expressed predominantly by dopaminergic neurons in the SN could refine this approach further, enabling precise delivery of anti-ferroptosis drugs while avoiding off-target effects, such as unintended disruption of iron metabolism in other brain regions. Balancing drug dosage is also critical, as low doses of weak chelators like PBT434 may reduce toxicity while maintaining therapeutic efficacy.

Stability and persistence of anti-ferroptosis drugs remain key challenges. Drugs must exhibit sufficient stability and durability to sustain therapeutic effects over the long term. Slow-release nanoparticle carriers or drugs with extended half-lives could address these issues, ensuring continuous inhibition of ferroptosis in the SN.

Finally, emerging targets such as apoferritin, iPLA2β, Nrf2, and HDACs offer new opportunities for therapeutic development. The gut microbiome is another area of interest, as studies suggest that LPC derived from gut microbiota can inhibit ferroptosis-related pathways in neurodegenerative diseases. Exploring these mechanisms could provide novel insights into the role of microbiota in neuroprotection for PD patients.

In summary, while targeting ferroptosis in PD is still in its early stages, it offers a promising therapeutic strategy. Addressing current challenges, such as BBB penetration, targeting efficiency, and drug stability, while leveraging advances in personalized medicine and emerging technologies, could pave the way for more effective treatments. Further research and clinical trials are essential to optimize these therapies and realize their potential in combating PD.

## Data Availability

Not applicable.

## References

[1] Tysnes O.B., Storstein A. (2017). Epidemiology of Parkinson’s disease. J. Neural Transm..

[2] Parkinson J. (1813). Mr. Parkinson’s Letter on the Committee of Apothecaries. Med. Phys. J..

[3] Kalinderi K., Papaliagkas V., Fidani L. (2019). Pharmacogenetics and levodopa induced motor complications. Int. J. Neurosci..

[4] Valldeoriola F., Torres V. (2023). Predicting impulse control disorder in Parkinson’s Disease: Is there a formula?. Eur. Neuropsychopharmacol..

[5] Dixon S.J., Lemberg K.M., Lamprecht M.R., Skouta R., Zaitsev E.M., Gleason C.E., Patel D.N., Bauer A.J., Cantley A.M., Yang W.S. (2012). Ferroptosis: An iron-dependent form of nonapoptotic cell death. Cell.

[6] Stockwell B.R., Friedmann Angeli J.P., Bayir H., Bush A.I., Conrad M., Dixon S.J., Fulda S., Gascón S., Hatzios S.K., Kagan V.E. (2017). Ferroptosis: A Regulated Cell Death Nexus Linking Metabolism, Redox Biology, and Disease. Cell.

[7] Zhang S., Xin W., Anderson G.J., Li R., Gao L., Chen S., Zhao J., Liu S. (2022). Double-edge sword roles of iron in driving energy production versus instigating ferroptosis. Cell Death Dis..

[8] Yu Y., Yan Y., Niu F., Wang Y., Chen X., Su G., Liu Y., Zhao X., Qian L., Liu P. (2021). Ferroptosis: A cell death connecting oxidative stress, inflammation and cardiovascular diseases. Cell Death Discov..

[9] Mahoney-Sánchez L., Bouchaoui H., Ayton S., Devos D., Duce J.A., Devedjian J.C. (2021). Ferroptosis and its potential role in the physiopathology of Parkinson’s Disease. Progress. Neurobiol..

[10] Nagatsu T., Sawada M. (2007). Biochemistry of postmortem brains in Parkinson’s disease: Historical overview and future prospects. Neuropsychiatric Disorders An Integrative Approach. Journal of Neural Transmission.

[11] Jiang H., Wang J., Rogers J., Xie J. (2017). Brain Iron Metabolism Dysfunction in Parkinson’s Disease. Mol. Neurobiol..

[12] Dexter D.T., Carter C.J., Wells F.R., Javoy-Agid F., Agid Y., Lees A., Jenner P., Marsden C.D. (1989). Basal lipid peroxidation in substantia nigra is increased in Parkinson’s disease. J. Neurochem..

[13] Melki R. (2015). Role of Different Alpha-Synuclein Strains in Synucleinopathies, Similarities with other Neurodegenerative Diseases. J. Park. Dis..

[14] Nikam S., Nikam P., Ahaley S.K., Sontakke A.V. (2009). Oxidative stress in Parkinson’s disease. Indian J. Clin. Biochem..

[15] Bose A., Beal M.F. (2016). Mitochondrial dysfunction in Parkinson’s disease. J. Neurochem..

[16] Whitton P.S. (2007). Inflammation as a causative factor in the aetiology of Parkinson’s disease. Br. J. Pharmacol..

[17] Zhang W., Zecca L., Wilson B., Ren H.W., Wang Y.J., Wang X.M., Hong J.S. (2013). Human neuromelanin: An endogenous microglial activator for dopaminergic neuron death. Front. Biosci. (Elite Ed).

[18] Sulzer D., Edwards R.H. (2019). The physiological role of α-synuclein and its relationship to Parkinson’s Disease. J. Neurochem..

[19] Golts N., Snyder H., Frasier M., Theisler C., Choi P., Wolozin B. (2002). Magnesium inhibits spontaneous and iron-induced aggregation of alpha-synuclein. J. Biol. Chem..

[20] Zhao Q., Tao Y., Zhao K., Ma Y., Xu Q., Liu C., Zhang S., Li D. (2023). Structural Insights of Fe(3+) Induced α-synuclein Fibrillation in Parkinson’s Disease. J. Mol. Biol..

[21] Zhou Z.D., Tan E.K. (2017). Iron regulatory protein (IRP)-iron responsive element (IRE) signaling pathway in human neurodegenerative diseases. Mol. Neurodegener..

[22] Saito Y., Kawashima A., Ruberu N.N., Fujiwara H., Koyama S., Sawabe M., Arai T., Nagura H., Yamanouchi H., Hasegawa M. (2003). Accumulation of phosphorylated alpha-synuclein in aging human brain. J. Neuropathol. Exp. Neurol..

[23] Han R., Wang Q., Xiong X., Chen X., Tu Z., Li B., Zhang F., Chen C., Pan M., Xu T. (2024). Deficiency of parkin causes neurodegeneration and accumulation of pathological α-synuclein in monkey models. J. Clin. Investig..

[24] Wang R., Wang Y., Qu L., Chen B., Jiang H., Song N., Xie J. (2019). Iron-induced oxidative stress contributes to α-synuclein phosphorylation and up-regulation via polo-like kinase 2 and casein kinase 2. Neurochem. Int..

[25] Davies P., Moualla D., Brown D.R. (2011). Alpha-synuclein is a cellular ferrireductase. PLoS ONE.

[26] Angelova P.R., Choi M.L., Berezhnov A.V., Horrocks M.H., Hughes C.D., De S., Rodrigues M., Yapom R., Little D., Dolt K.S. (2020). Alpha synuclein aggregation drives ferroptosis: An interplay of iron, calcium and lipid peroxidation. Cell Death Differ..

[27] Costa I., Barbosa D.J., Benfeito S., Silva V., Chavarria D., Borges F., Remião F., Silva R. (2023). Molecular mechanisms of ferroptosis and their involvement in brain diseases. Pharmacol. Ther..

[28] Mahoney-Sanchez L., Bouchaoui H., Boussaad I., Jonneaux A., Timmerman K., Berdeaux O., Ayton S., Krüger R., Duce J.A., Devos D. (2022). Alpha synuclein determines ferroptosis sensitivity in dopaminergic neurons via modulation of ether-phospholipid membrane composition. Cell Rep..

[29] Shamoto-Nagai M., Hisaka S., Naoi M., Maruyama W. (2018). Modification of α-synuclein by lipid peroxidation products derived from polyunsaturated fatty acids promotes toxic oligomerization: Its relevance to Parkinson disease. J. Clin. Biochem. Nutr..

[30] Ganguly U., Singh S., Bir A., Ghosh A., Chakrabarti S.S., Saini R.V., Saso L., Bisaglia M., Chakrabarti S. (2024). Alpha-synuclein interaction with mitochondria is the final mechanism of ferroptotic death induced by erastin in SH-SY5Y cells. Free Radic. Res..

[31] Wu L., Liu M., Liang J., Li N., Yang D., Cai J., Zhang Y., He Y., Chen Z., Ma T. (2021). Ferroptosis as a New Mechanism in Parkinson’s Disease Therapy Using Traditional Chinese Medicine. Front. Pharmacol..

[32] Vallerga C.L., Zhang F., Fowdar J., McRae A.F., Qi T., Nabais M.F., Zhang Q., Kassam I., Henders A.K., Wallace L. (2020). Analysis of DNA methylation associates the cystine-glutamate antiporter SLC7A11 with risk of Parkinson’s disease. Nat. Commun..

[33] Aoyama K., Nakaki T. (2015). Glutathione in Cellular Redox Homeostasis: Association with the Excitatory Amino Acid Carrier 1 (EAAC1). Molecules.

[34] Battaglia A.M., Chirillo R., Aversa I., Sacco A., Costanzo F., Biamonte F. (2020). Ferroptosis and Cancer: Mitochondria Meet the “Iron Maiden” Cell Death. Cells.

[35] Cao J., Chen X., Jiang L., Lu B., Yuan M., Zhu D., Zhu H., He Q., Yang B., Ying M. (2020). DJ-1 suppresses ferroptosis through preserving the activity of S-adenosyl homocysteine hydrolase. Nat. Commun..

[36] Mao C., Liu X., Zhang Y., Lei G., Yan Y., Lee H., Koppula P., Wu S., Zhuang L., Fang B. (2021). DHODH-mediated ferroptosis defence is a targetable vulnerability in cancer. Nature.

[37] Shimada K., Skouta R., Kaplan A., Yang W.S., Hayano M., Dixon S.J., Brown L.M., Valenzuela C.A., Wolpaw A.J., Stockwell B.R. (2016). Global survey of cell death mechanisms reveals metabolic regulation of ferroptosis. Nat. Chem. Biol..

[38] Kraft V.A.N., Bezjian C.T., Pfeiffer S., Ringelstetter L., Müller C., Zandkarimi F., Merl-Pham J., Bao X., Anastasov N., Kössl J. (2020). GTP Cyclohydrolase 1/Tetrahydrobiopterin Counteract Ferroptosis through Lipid Remodeling. ACS Cent. Sci..

[39] Jian X., Zhao G., Chen H., Wang Y., Li J., Xie L., Li B. (2022). Revealing a novel contributing landscape of ferroptosis-related genes in Parkinson’s disease. Comput. Struct. Biotechnol. J..

[40] Chen Y.P., Yu S.H., Zhang G.H., Hou Y.B., Gu X.J., Ou R.W., Shen Y., Song W., Chen X.P., Zhao B. (2022). The mutation spectrum of Parkinson-disease-related genes in early-onset Parkinson’s disease in ethnic Chinese. Eur. J. Neurol..

[41] Friedmann Angeli J.P., Schneider M., Proneth B., Tyurina Y.Y., Tyurin V.A., Hammond V.J., Herbach N., Aichler M., Walch A., Eggenhofer E. (2014). Inactivation of the ferroptosis regulator Gpx4 triggers acute renal failure in mice. Nat. Cell Biol..

[42] Wu H., Wang F., Ta N., Zhang T., Gao W. (2021). The Multifaceted Regulation of Mitochondria in Ferroptosis. Life.

[43] Hare D.J., Double K.L. (2016). Iron and dopamine: A toxic couple. Brain.

[44] Levi S., Corsi B., Bosisio M., Invernizzi R., Volz A., Sanford D., Arosio P., Drysdale J. (2001). A human mitochondrial ferritin encoded by an intronless gene. J. Biol. Chem..

[45] Do Van B., Gouel F., Jonneaux A., Timmerman K., Gelé P., Pétrault M., Bastide M., Laloux C., Moreau C., Bordet R. (2016). Ferroptosis, a newly characterized form of cell death in Parkinson’s disease that is regulated by PKC. Neurobiol. Dis..

[46] Avcı B., Günaydın C., Güvenç T., Yavuz C.K., Kuruca N., Bilge S.S. (2021). Idebenone Ameliorates Rotenone-Induced Parkinson’s Disease in Rats Through Decreasing Lipid Peroxidation. Neurochem. Res..

[47] Yagoda N., von Rechenberg M., Zaganjor E., Bauer A.J., Yang W.S., Fridman D.J., Wolpaw A.J., Smukste I., Peltier J.M., Boniface J.J. (2007). RAS-RAF-MEK-dependent oxidative cell death involving voltage-dependent anion channels. Nature.

[48] Rajendran M., Queralt-Martín M., Gurnev P.A., Rosencrans W.M., Rovini A., Jacobs D., Abrantes K., Hoogerheide D.P., Bezrukov S.M., Rostovtseva T.K. (2022). Restricting α-synuclein transport into mitochondria by inhibition of α-synuclein-VDAC complexation as a potential therapeutic target for Parkinson’s disease treatment. Cell. Mol. Life Sci..

[49] Scheiblich H., Eikens F., Wischhof L., Opitz S., Jüngling K., Cserép C., Schmidt S.V., Lambertz J., Bellande T., Pósfai B. (2024). Microglia rescue neurons from aggregate-induced neuronal dysfunction and death through tunneling nanotubes. Neuron.

[50] Joers V., Tansey M.G., Mulas G., Carta A.R. (2017). Microglial phenotypes in Parkinson’s disease and animal models of the disease. Prog. Neurobiol..

[51] Xu L., He D., Bai Y. (2016). Microglia-Mediated Inflammation and Neurodegenerative Disease. Mol. Neurobiol..

[52] Urrutia P., Aguirre P., Esparza A., Tapia V., Mena N.P., Arredondo M., González-Billault C., Núñez M.T. (2013). Inflammation alters the expression of DMT1, FPN1 and hepcidin, and it causes iron accumulation in central nervous system cells. J. Neurochem..

[53] Ryan S.K., Zelic M., Han Y., Teeple E., Chen L., Sadeghi M., Shankara S., Guo L., Li C., Pontarelli F. (2023). Microglia ferroptosis is regulated by SEC24B and contributes to neurodegeneration. Nat. Neurosci..

[54] Zecca L., Casella L., Albertini A., Bellei C., Zucca F.A., Engelen M., Zadlo A., Szewczyk G., Zareba M., Sarna T. (2008). Neuromelanin can protect against iron-mediated oxidative damage in system modeling iron overload of brain aging and Parkinson’s disease. J. Neurochem..

[55] Li J., Yang J., Zhao P., Li S., Zhang R., Zhang X., Liu D., Zhang B. (2012). Neuromelanin enhances the toxicity of α-synuclein in SK-N-SH cells. J. Neural Transm..

[56] Li L., Fang C.J., Ryan J.C., Niemi E.C., Lebrón J.A., Björkman P.J., Arase H., Torti F.M., Torti S.V., Nakamura M.C. (2010). Binding and uptake of H-ferritin are mediated by human transferrin receptor-1. Proc. Natl. Acad. Sci. USA.

[57] Tian Y., Lu J., Hao X., Li H., Zhang G., Liu X., Li X., Zhao C., Kuang W., Chen D. (2020). FTH1 Inhibits Ferroptosis Through Ferritinophagy in the 6-OHDA Model of Parkinson’s Disease. Neurotherapeutics.

[58] Jiang X., Zhou T., Bai R., Xie Y. (2020). Hydroxypyridinone-Based Iron Chelators with Broad-Ranging Biological Activities. J. Med. Chem..

[59] Yanatori I., Kishi F. (2019). DMT1 and iron transport. Free Radic. Biol. Med..

[60] Bórquez D.A., Urrutia P.J. (2024). Iron regulatory protein 1: The deadly switch of ferroptosis. Neural Regen. Res..

[61] Su Y., Jiao Y., Cai S., Xu Y., Wang Q., Chen X. (2024). The molecular mechanism of ferroptosis and its relationship with Parkinson’s disease. Brain Res. Bull..

[62] Zeng X., An H., Yu F., Wang K., Zheng L., Zhou W., Bao Y., Yang J., Shen N., Huang D. (2021). Benefits of Iron Chelators in the Treatment of Parkinson’s Disease. Neurochem. Res..

[63] Chen X., Yu C., Kang R., Tang D. (2020). Iron Metabolism in Ferroptosis. Front. Cell Dev. Biol..

[64] Devos D., Moreau C., Devedjian J.C., Kluza J., Petrault M., Laloux C., Jonneaux A., Ryckewaert G., Garçon G., Rouaix N. (2014). Targeting chelatable iron as a therapeutic modality in Parkinson’s disease. Antioxid. Redox Signal..

[65] Martin-Bastida A., Ward R.J., Newbould R., Piccini P., Sharp D., Kabba C., Patel M.C., Spino M., Connelly J., Tricta F. (2017). Brain iron chelation by deferiprone in a phase 2 randomised double-blinded placebo controlled clinical trial in Parkinson’s disease. Sci. Rep..

[66] Ayton S., Lei P., McLean C., Bush A.I., Finkelstein D.I. (2016). Transferrin protects against Parkinsonian neurotoxicity and is deficient in Parkinson’s substantia nigra. Signal Transduct. Target. Ther..

[67] Finkelstein D.I., Billings J.L., Adlard P.A., Ayton S., Sedjahtera A., Masters C.L., Wilkins S., Shackleford D.M., Charman S.A., Bal W. (2017). The novel compound PBT434 prevents iron mediated neurodegeneration and alpha-synuclein toxicity in multiple models of Parkinson’s disease. Acta Neuropathol. Commun..

[68] Zheng H., Gal S., Weiner L.M., Bar-Am O., Warshawsky A., Fridkin M., Youdim M.B. (2005). Novel multifunctional neuroprotective iron chelator-monoamine oxidase inhibitor drugs for neurodegenerative diseases: In vitro studies on antioxidant activity, prevention of lipid peroxide formation and monoamine oxidase inhibition. J. Neurochem..

[69] Shachar D.B., Kahana N., Kampel V., Warshawsky A., Youdim M.B. (2004). Neuroprotection by a novel brain permeable iron chelator, VK-28, against 6-hydroxydopamine lession in rats. Neuropharmacology.

[70] Zhu W., Xie W., Pan T., Xu P., Fridkin M., Zheng H., Jankovic J., Youdim M.B., Le W. (2007). Prevention and restoration of lactacystin-induced nigrostriatal dopamine neuron degeneration by novel brain-permeable iron chelators. FASEB J..

[71] Zheng H., Weiner L.M., Bar-Am O., Epsztejn S., Cabantchik Z.I., Warshawsky A., Youdim M.B., Fridkin M. (2005). Design, synthesis, and evaluation of novel bifunctional iron-chelators as potential agents for neuroprotection in Alzheimer’s, Parkinson’s, and other neurodegenerative diseases. Bioorganic Med. Chem..

[72] Asao M. (1979). Clioquinol and S.M.O.N.: Reanalysis of original data. Lancet.

[73] Shi L., Huang C., Luo Q., Xia Y., Liu W., Zeng W., Cheng A., Shi R., Zhengli C. (2020). Clioquinol improves motor and non-motor deficits in MPTP-induced monkey model of Parkinson’s disease through AKT/mTOR pathway. Aging.

[74] Du X.-X., Xu H.-M., Jiang H., Song N., Wang J., Xie J.-X. (2012). Curcumin protects nigral dopaminergic neurons by iron-chelation in the 6-hydroxydopamine rat model of Parkinson’s disease. Neurosci. Bull..

[75] Yazdanparast M.M.a.R. (2013). Protective Effects of Flavonoid Baicalein against Menadione-Induced Damage in SK-N-MC Cells. CellBio.

[76] Lee E., Park H.R., Ji S.T., Lee Y., Lee J. (2014). Baicalein attenuates astroglial activation in the 1-methyl-4-phenyl-1,2,3,4-tetrahydropyridine-induced Parkinson’s disease model by downregulating the activations of nuclear factor-κB, ERK, and JNK. J. Neurosci. Res..

[77] Li X., Zhang G., Nie Q., Wu T., Jiao L., Zheng M., Wan X., Li Y., Wu S., Jiang B. (2017). Baicalein blocks α-synuclein secretion from SN4741 cells and facilitates α-synuclein polymerization to big complex. Neurosci. Lett..

[78] Song L.M., Xiao Z.X., Zhang N., Yu X.Q., Cui W., Xie J.X., Xu H.M. (2021). Apoferritin improves motor deficits in MPTP-treated mice by regulating brain iron metabolism and ferroptosis. iScience.

[79] Berry T.M., Moustafa A.A. (2023). A novel treatment strategy to prevent Parkinson’s disease: Focus on iron regulatory protein 1 (IRP1). Int. J. Neurosci..

[80] Li X., Si W., Li Z., Tian Y., Liu X., Ye S., Huang Z., Ji Y., Zhao C., Hao X. (2021). miR-335 promotes ferroptosis by targeting ferritin heavy chain 1 in in vivo and in vitro models of Parkinson’s disease. Int. J. Mol. Med..

[81] Asci R., Vallefuoco F., Andolfo I., Bruno M., De Falco L., Iolascon A. (2013). Trasferrin receptor 2 gene regulation by microRNA 221 in SH-SY5Y cells treated with MPP⁺ as Parkinson’s disease cellular model. Neurosci. Res..

[82] Iqbal S., Jabeen F., Kahwa I., Omara T. (2023). Suberosin Alleviates Thiazolidinedione-Induced Cardiomyopathy in Diabetic Rats by Inhibiting Ferroptosis via Modulation of ACSL4-LPCAT3 and PI3K-AKT Signaling Pathways. Cardiovasc. Toxicol..

[83] Chu B., Kon N., Chen D., Li T., Liu T., Jiang L., Song S., Tavana O., Gu W. (2019). ALOX12 is required for p53-mediated tumour suppression through a distinct ferroptosis pathway. Nat. Cell Biol..

[84] Chou V.P., Ko N., Holman T.R., Manning-Boğ A.B. (2014). Gene-environment interaction models to unmask susceptibility mechanisms in Parkinson’s disease. J. Vis. Exp..

[85] Koppula P., Zhuang L., Gan B. (2021). Cytochrome P450 reductase (POR) as a ferroptosis fuel. Protein Cell.

[86] Taghizadeh M., Tamtaji O.R., Dadgostar E., Daneshvar Kakhaki R., Bahmani F., Abolhassani J., Aarabi M.H., Kouchaki E., Memarzadeh M.R., Asemi Z. (2017). The effects of omega-3 fatty acids and vitamin E co-supplementation on clinical and metabolic status in patients with Parkinson’s disease: A randomized, double-blind, placebo-controlled trial. Neurochem. Int..

[87] Southon A., Szostak K., Acevedo K.M., Dent K.A., Volitakis I., Belaidi A.A., Barnham K.J., Crouch P.J., Ayton S., Donnelly P.S. (2020). Cu(II) (atsm) inhibits ferroptosis: Implications for treatment of neurodegenerative disease. Br. J. Pharmacol..

[88] Zilka O., Poon J.F., Pratt D.A. (2021). Radical-Trapping Antioxidant Activity of Copper and Nickel Bis(Thiosemicarbazone) Complexes Underlies Their Potency as Inhibitors of Ferroptotic Cell Death. J. Am. Chem. Soc..

[89] Jiang X., Wu K., Ye X.Y., Xie T., Zhang P., Blass B.E., Bai R. (2023). Novel druggable mechanism of Parkinson’s disease: Potential therapeutics and underlying pathogenesis based on ferroptosis. Med. Res. Rev..

[90] Skouta R., Dixon S.J., Wang J., Dunn D.E., Orman M., Shimada K., Rosenberg P.A., Lo D.C., Weinberg J.M., Linkermann A. (2014). Ferrostatins inhibit oxidative lipid damage and cell death in diverse disease models. J. Am. Chem. Soc..

[91] Devisscher L., Van Coillie S., Hofmans S., Van Rompaey D., Goossens K., Meul E., Maes L., De Winter H., Van Der Veken P., Vandenabeele P. (2018). Discovery of Novel, Drug-Like Ferroptosis Inhibitors with in Vivo Efficacy. J. Med. Chem..

[92] Ji H.L., Zhang Y.F., Zhang N.Y., Wang K.M., Meng N., Zhang J., Jiang C.S. (2024). Design, synthesis, and evaluation of formylpiperazine analogs of Ferrostatin-1 as novel improved ferroptosis inhibitors. Bioorganic Med. Chem..

[93] Morrow J.P., Mazrad Z.A.I., Warne N.M., Ayton S., Bush A.I., Kempe K. (2024). Schiff-Base Cross-Linked Poly(2-oxazoline) Micelle Drug Conjugates Possess Antiferroptosis Activity in Numerous In Vitro Cell Models. Biomacromolecules.

[94] Kou D., Gao Y., Li C., Zhou D., Lu K., Wang N., Zhang R., Yang Z., Zhou Y., Chen L. (2023). Intranasal Pathway for Nanoparticles to Enter the Central Nervous System. Nano Lett..

[95] Goertsen D., Flytzanis N.C., Goeden N., Chuapoco M.R., Cummins A., Chen Y., Fan Y., Zhang Q., Sharma J., Duan Y. (2022). AAV capsid variants with brain-wide transgene expression and decreased liver targeting after intravenous delivery in mouse and marmoset. Nat. Neurosci..

[96] Yin W., Ma H., Qu Y., Ren J., Sun Y., Guo Z.N., Yang Y. (2025). Exosomes: The next-generation therapeutic platform for ischemic stroke. Neural Regen. Res..

[97] Doll S., Proneth B., Tyurina Y.Y., Panzilius E., Kobayashi S., Ingold I., Irmler M., Beckers J., Aichler M., Walch A. (2017). ACSL4 dictates ferroptosis sensitivity by shaping cellular lipid composition. Nat. Chem. Biol..

[98] Yuan H., Li X., Zhang X., Kang R., Tang D. (2016). Identification of ACSL4 as a biomarker and contributor of ferroptosis. Biochem. Biophys. Res. Commun..

[99] Kagan V.E., Mao G., Qu F., Angeli J.P., Doll S., Croix C.S., Dar H.H., Liu B., Tyurin V.A., Ritov V.B. (2017). Oxidized arachidonic and adrenic PEs navigate cells to ferroptosis. Nat. Chem. Biol..

[100] Huang Q., Ru Y., Luo Y., Luo X., Liu D., Ma Y., Zhou X., Linghu M., Xu W., Gao F. (2024). Identification of a targeted ACSL4 inhibitor to treat ferroptosis-related diseases. Sci. Adv..

[101] Yue M., Wei J., Chen W., Hong D., Chen T., Fang X. (2022). Neurotrophic Role of the Next-Generation Probiotic Strain L. lactis MG1363-pMG36e-GLP-1 on Parkinson’s Disease via Inhibiting Ferroptosis. Nutrients.

[102] Reed A., Ichu T.A., Milosevich N., Melillo B., Schafroth M.A., Otsuka Y., Scampavia L., Spicer T.P., Cravatt B.F. (2022). LPCAT3 Inhibitors Remodel the Polyunsaturated Phospholipid Content of Human Cells and Protect from Ferroptosis. ACS Chem. Biol..

[103] Li K., Wang M., Huang Z.H., Wang M., Sun W.Y., Kurihara H., Huang R.T., Wang R., Huang F., Liang L. (2023). ALOX5 inhibition protects against dopaminergic neurons undergoing ferroptosis. Pharmacol. Res..

[104] Razavi S.M., Khayatan D., Arab Z.N., Momtaz S., Zare K., Jafari R.M., Dehpour A.R., Abdolghaffari A.H. (2021). Licofelone, a potent COX/5-LOX inhibitor and a novel option for treatment of neurological disorders. Prostaglandins Other Lipid Mediat..

[105] Yang W.S., Kim K.J., Gaschler M.M., Patel M., Shchepinov M.S., Stockwell B.R. (2016). Peroxidation of polyunsaturated fatty acids by lipoxygenases drives ferroptosis. Proc. Natl. Acad. Sci. USA.

[106] Angelova P.R., Horrocks M.H., Klenerman D., Gandhi S., Abramov A.Y., Shchepinov M.S. (2015). Lipid peroxidation is essential for α-synuclein-induced cell death. J. Neurochem..

[107] Ke M., Chong C.M., Zeng H., Huang M., Huang Z., Zhang K., Cen X., Lu J.H., Yao X., Qin D. (2020). Azoramide protects iPSC-derived dopaminergic neurons with PLA2G6 D331Y mutation through restoring ER function and CREB signaling. Cell Death Dis..

[108] Chen D., Chu B., Yang X., Liu Z., Jin Y., Kon N., Rabadan R., Jiang X., Stockwell B.R., Gu W. (2021). iPLA2β-mediated lipid detoxification controls p53-driven ferroptosis independent of GPX4. Nat. Commun..

[109] Yeh T.H., Liu H.F., Chiu C.C., Cheng M.L., Huang G.J., Huang Y.C., Liu Y.C., Huang Y.Z., Lu C.S., Chen Y.C. (2021). PLA2G6 mutations cause motor dysfunction phenotypes of young-onset dystonia-parkinsonism type 14 and can be relieved by DHA treatment in animal models. Exp. Neurol..

[110] Liu J., Tan J., Tang B., Guo J. (2024). Unveiling the role of iPLA(2)β in neurodegeneration: From molecular mechanisms to advanced therapies. Pharmacol. Res..

[111] Mischley L.K., Lau R.C., Shankland E.G., Wilbur T.K., Padowski J.M. (2017). Phase IIb Study of Intranasal Glutathione in Parkinson’s Disease. J. Park. Dis..

[112] Sechi G., Deledda M.G., Bua G., Satta W.M., Deiana G.A., Pes G.M., Rosati G. (1996). Reduced intravenous glutathione in the treatment of early Parkinson’s disease. Prog. Neuro-Psychopharmacol. Biol. Psychiatry.

[113] Coles L.D., Tuite P.J., Öz G., Mishra U.R., Kartha R.V., Sullivan K.M., Cloyd J.C., Terpstra M. (2018). Repeated-Dose Oral N-Acetylcysteine in Parkinson’s Disease: Pharmacokinetics and Effect on Brain Glutathione and Oxidative Stress. J. Clin. Pharmacol..

[114] Holmay M.J., Terpstra M., Coles L.D., Mishra U., Ahlskog M., Öz G., Cloyd J.C., Tuite P.J. (2013). N-Acetylcysteine boosts brain and blood glutathione in Gaucher and Parkinson diseases. Clin. Neuropharmacol..

[115] Ingold I., Berndt C., Schmitt S., Doll S., Poschmann G., Buday K., Roveri A., Peng X., Porto Freitas F., Seibt T. (2018). Selenium Utilization by GPX4 Is Required to Prevent Hydroperoxide-Induced Ferroptosis. Cell.

[116] Mischley L.K., Allen J., Bradley R. (2012). Coenzyme Q10 deficiency in patients with Parkinson’s disease. J. Neurol. Sci..

[117] Bai L., Yan F., Deng R., Gu R., Zhang X., Bai J. (2021). Thioredoxin-1 Rescues MPP(+)/MPTP-Induced Ferroptosis by Increasing Glutathione Peroxidase 4. Mol. Neurobiol..

[118] Gao Z., Wang G., Chen Y., Yuan W., Cai J., Feng A., Fang J., Xu Q., Wu X. (2024). Total flavonoids of Astragalus membranaceus protect against 1-methyl-4-phenyl-1,2,3,6-tetrahydropyridine-induced neurotoxicity in mice by inhibiting ferroptosis through SLC7A11/GPX-4 signaling pathway. Food Sci. Hum. Wellness.

[119] Coe S., Andreoli D., George M., Collett J., Reed A., Cossington J., Izadi H., Dixon A., Mansoubi M., Dawes H. (2022). A feasibility study to determine whether the daily consumption of flavonoid-rich pure cocoa has the potential to reduce fatigue and fatigability in people with Parkinson’s (pwP). Clin. Nutr. ESPEN.

[120] Pang H., Xue W., Shi A., Li M., Li Y., Cao G., Yan B., Dong F., Xiao W., He G. (2016). Multiple-Ascending-Dose Pharmacokinetics and Safety Evaluation of Baicalein Chewable Tablets in Healthy Chinese Volunteers. Clin. Drug Investig..

[121] Adinolfi S., Patinen T., Jawahar Deen A., Pitkänen S., Härkönen J., Kansanen E., Küblbeck J., Levonen A.L. (2023). The KEAP1-NRF2 pathway: Targets for therapy and role in cancer. Redox Biol..

[122] Impellizzeri D., Cordaro M., Siracusa R., Fusco R., Peritore A.F., Gugliandolo E., Genovese T., Crupi R., Interdonato L., Evangelista M. (2023). Molecular targets for anti-oxidative protection of açaí berry against diabetes myocardial ischemia/reperfusion injury. Free Radic. Res..

[123] Thapa K., Khan H., Kanojia N., Singh T.G., Kaur A., Kaur G. (2022). Therapeutic Insights on Ferroptosis in Parkinson’s disease. Eur. J. Pharmacol..

[124] Osburn W.O., Wakabayashi N., Misra V., Nilles T., Biswal S., Trush M.A., Kensler T.W. (2006). Nrf2 regulates an adaptive response protecting against oxidative damage following diquat-mediated formation of superoxide anion. Arch. Biochem. Biophys..

[125] Kerins M.J., Ooi A. (2018). The Roles of NRF2 in Modulating Cellular Iron Homeostasis. Antioxid. Redox Signal..

[126] Liu H., Zhang T.A., Zhang W.Y., Huang S.R., Hu Y., Sun J. (2023). Rhein attenuates cerebral ischemia-reperfusion injury via inhibition of ferroptosis through NRF2/SLC7A11/GPX4 pathway. Exp. Neurol..

[127] Kovac S., Angelova P.R., Holmström K.M., Zhang Y., Dinkova-Kostova A.T., Abramov A.Y. (2015). Nrf2 regulates ROS production by mitochondria and NADPH oxidase. Biochim. Biophys. Acta.

[128] Masaki Y., Izumi Y., Matsumura A., Akaike A., Kume T. (2017). Protective effect of Nrf2-ARE activator isolated from green perilla leaves on dopaminergic neuronal loss in a Parkinson’s disease model. Eur. J. Pharmacol..

[129] Liu L., Yang S., Wang H. (2021). α-Lipoic acid alleviates ferroptosis in the MPP^+^-induced PC12 cells via activating the PI3K/Akt/Nrf2 pathway. Cell Biol. Int..

[130] Mao J., Gao H., Bai W., Zeng H., Ren Y., Liu Y., Yang X. (2021). Lipoic acid alleviates LPS-evoked PC12 cell damage by targeting p53 and inactivating the NF-κB pathway. Acta Neurobiol. Exp..

[131] Li J., Yang D., Li Z., Zhao M., Wang D., Sun Z., Wen P., Dai Y., Gou F., Ji Y. (2023). PINK1/Parkin-mediated mitophagy in neurodegenerative diseases. Ageing Res. Rev..

[132] Han Z., Wang B., Wen Y.Q., Li Y.N., Feng C.X., Ding X.S., Shen Y., Yang Q., Gao L. (2024). Acteoside alleviates lipid peroxidation by enhancing Nrf2-mediated mitophagy to inhibit ferroptosis for neuroprotection in Parkinson’s disease. Free Radic. Biol. Med..

[133] Zhao M., Wang B., Zhang C., Su Z., Guo B., Zhao Y., Zheng R. (2021). The DJ1-Nrf2-STING axis mediates the neuroprotective effects of Withaferin A in Parkinson’s disease. Cell Death Differ..

[134] Quek H., Luff J., Cheung K., Kozlov S., Gatei M., Lee C.S., Bellingham M.C., Noakes P.G., Lim Y.C., Barnett N.L. (2017). A rat model of ataxia-telangiectasia: Evidence for a neurodegenerative phenotype. Hum. Mol. Genet..

[135] Nazmi A., Field R.H., Griffin E.W., Haugh O., Hennessy E., Cox D., Reis R., Tortorelli L., Murray C.L., Lopez-Rodriguez A.B. (2019). Chronic neurodegeneration induces type I interferon synthesis via STING, shaping microglial phenotype and accelerating disease progression. Glia.

[136] Takahashi M., Yamada T. (1999). Viral etiology for Parkinson’s disease--a possible role of influenza A virus infection. Jpn. J. Infect. Dis..

[137] Holm C.K., Rahbek S.H., Gad H.H., Bak R.O., Jakobsen M.R., Jiang Z., Hansen A.L., Jensen S.K., Sun C., Thomsen M.K. (2016). Influenza A virus targets a cGAS-independent STING pathway that controls enveloped RNA viruses. Nat. Commun..

[138] Cleren C., Yang L., Lorenzo B., Calingasan N.Y., Schomer A., Sireci A., Wille E.J., Beal M.F. (2008). Therapeutic effects of coenzyme Q10 (CoQ10) and reduced CoQ10 in the MPTP model of Parkinsonism. J. Neurochem..

[139] Storch A., Jost W.H., Vieregge P., Spiegel J., Greulich W., Durner J., Müller T., Kupsch A., Henningsen H., Oertel W.H. (2007). Randomized, double-blind, placebo-controlled trial on symptomatic effects of coenzyme Q(10) in Parkinson disease. Arch. Neurol..

[140] Müller T., Büttner T., Gholipour A.F., Kuhn W. (2003). Coenzyme Q10 supplementation provides mild symptomatic benefit in patients with Parkinson’s disease. Neurosci. Lett..

[141] Erb M., Hoffmann-Enger B., Deppe H., Soeberdt M., Haefeli R.H., Rummey C., Feurer A., Gueven N. (2012). Features of idebenone and related short-chain quinones that rescue ATP levels under conditions of impaired mitochondrial complex I. PLoS ONE.

[142] Montenegro L., Turnaturi R., Parenti C., Pasquinucci L. (2018). Idebenone: Novel Strategies to Improve Its Systemic and Local Efficacy. Nanomaterials.

[143] Yan J., Sun W., Shen M., Zhang Y., Jiang M., Liu A., Ma H., Lai X., Wu J. (2022). Idebenone improves motor dysfunction, learning and memory by regulating mitophagy in MPTP-treated mice. Cell Death Discov..

[144] Fang Y., Tan Q., Zhou H., Gu Q., Xu J. (2022). Discovery of novel diphenylbutene derivative ferroptosis inhibitors as neuroprotective agents. Eur. J. Med. Chem..

[145] Oveisgharan S., Yu L., Barnes L.L., Agrawal S., Schneider J.A., Bennett D.A., Buchman A.S. (2022). Association of Statins With Cerebral Atherosclerosis and Incident Parkinsonism in Older Adults. Neurology.

[146] Kimura Y., Mochizuki H. (2024). Targeting Glucagon-Like Peptide 1 Signaling: A Potential Disease Modifying Therapy for Parkinson’s Disease. Mov. Disord..

[147] Marathe C.S., Rayner C.K., Jones K.L., Horowitz M. (2013). Glucagon-like peptides 1 and 2 in health and disease: A review. Peptides.

[148] Cryan J.F., O’Riordan K.J., Cowan C.S.M., Sandhu K.V., Bastiaanssen T.F.S., Boehme M., Codagnone M.G., Cussotto S., Fulling C., Golubeva A.V. (2019). The Microbiota-Gut-Brain Axis. Physiol. Rev..

[149] Lubomski M., Tan A.H., Lim S.Y., Holmes A.J., Davis R.L., Sue C.M. (2020). Parkinson’s disease and the gastrointestinal microbiome. J. Neurol..

[150] Zha X., Liu X., Wei M., Huang H., Cao J., Liu S., Bian X., Zhang Y., Xiao F., Xie Y. (2024). Microbiota-derived lysophosphatidylcholine alleviates Alzheimer’s disease pathology via suppressing ferroptosis. Cell Metab..

[151] Zhang W., Chen H., Ding L., Gong J., Zhang M., Guo W., Xu P., Li S., Zhang Y. (2021). Trojan Horse Delivery of 4,4′-Dimethoxychalcone for Parkinsonian Neuroprotection. Adv. Sci..

